# ATR represents a therapeutic vulnerability in clear cell renal cell carcinoma

**DOI:** 10.1172/jci.insight.156087

**Published:** 2022-12-22

**Authors:** Philipp Seidel, Anne Rubarth, Kyra Zodel, Asin Peighambari, Felix Neumann, Yannick Federkiel, Hsin Huang, Rouven Hoefflin, Mojca Adlesic, Christian Witt, David J. Hoffmann, Patrick Metzger, Ralph K. Lindemann, Frank T. Zenke, Christoph Schell, Melanie Boerries, Dominik von Elverfeldt, Wilfried Reichardt, Marie Follo, Joachim Albers, Ian J. Frew

**Affiliations:** 1 Department of Internal Medicine I, Hematology, Oncology and Stem Cell Transplantation, Faculty of Medicine, Medical Center – University of Freiburg, Freiburg, Germany.; 2 Translational Innovation Platform Oncology and Immuno-Oncology, the Healthcare Business of Merck KGaA, Darmstadt, Germany.; 3 Institute of Medical Bioinformatics and; 4 Institute for Surgical Pathology, Faculty of Medicine, Medical Center – University of Freiburg, Freiburg, Germany.; 5 German Cancer Consortium (DKTK), German Cancer Research Center (DKFZ) Partner Site Freiburg, Freiburg, Germany.; 6 Comprehensive Cancer Center Freiburg (CCCF) and; 7 Medical Physics, Department of Radiology, Faculty of Medicine, Medical Center – University of Freiburg, Freiburg, Germany.; 8 Signalling Research Center BIOSS, University of Freiburg, Freiburg, Germany.

**Keywords:** Oncology, DNA repair, Urology

## Abstract

Metastatic clear cell renal cell carcinomas (ccRCCs) are resistant to DNA-damaging chemotherapies, limiting therapeutic options for patients whose tumors are resistant to tyrosine kinase inhibitors and/or immune checkpoint therapies. Here we show that mouse and human ccRCCs were frequently characterized by high levels of endogenous DNA damage and that cultured ccRCC cells exhibited intact cellular responses to chemotherapy-induced DNA damage. We identify that pharmacological inhibition of the DNA damage–sensing kinase ataxia telangiectasia and Rad3-related protein (ATR) with the orally administered, potent, and selective drug M4344 (gartisertib) induced antiproliferative effects in ccRCC cells. This effect was due to replication stress and accumulation of DNA damage in S phase. In some cells, DNA damage persisted into subsequent G_2_/M and G_1_ phases, leading to the frequent accumulation of micronuclei. Daily single-agent treatment with M4344 inhibited the growth of ccRCC xenograft tumors. M4344 synergized with chemotherapeutic drugs including cisplatin and carboplatin and the poly(ADP-ribose) polymerase inhibitor olaparib in mouse and human ccRCC cells. Weekly M4344 plus cisplatin treatment showed therapeutic synergy in ccRCC xenografts and was efficacious in an autochthonous mouse ccRCC model. These studies identify ATR inhibition as a potential novel therapeutic option for ccRCC.

## Graphical abstract

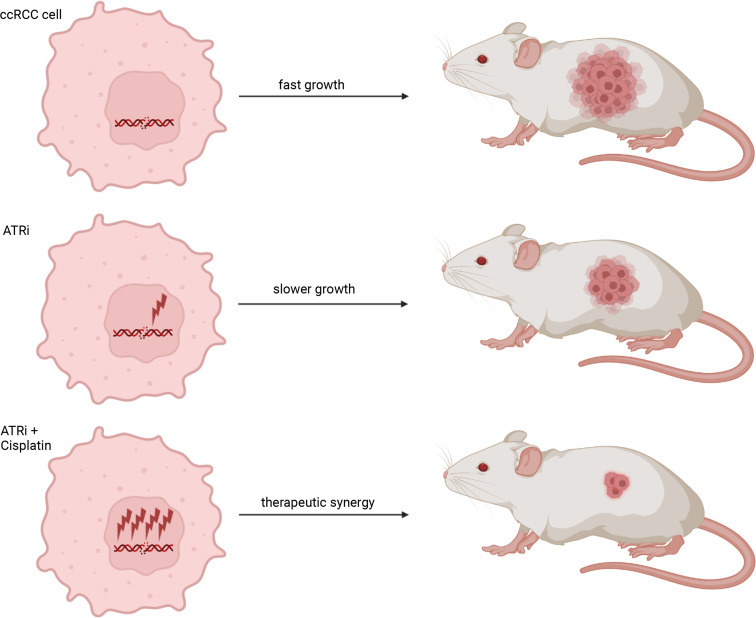

## Introduction

Current therapies for metastatic clear cell renal cell carcinoma (ccRCC) involve immunomodulatory therapy targeting the programmed cell death 1 (PD-1)/programmed cell death ligand 1 and/or cytotoxic T lymphocyte–associated protein 4 immune checkpoints, in combination with a number of tyrosine kinase inhibitors (TKIs) that inhibit VEGF and other signaling pathways (1, 2). These therapeutic options have improved tumor responses and overall patient survival, but primary and secondary resistance as well as therapeutic intolerance remain common problems that frequently lead to treatment failure. Many cases of metastatic ccRCC ultimately remain incurable. It would be desirable to develop new therapies that target different biological sensitivities of ccRCC tumors. Here we describe a potentially novel sensitivity of ccRCC cells to inhibition of the DNA damage signaling protein kinase ataxia telangiectasia and Rad3-related protein (ATR).

Up to 92% of sporadic ccRCC tumors harbor biallelic inactivation of the von Hippel-Lindau (*VHL*) tumor suppressor gene due to loss of 1 copy of chromosome 3p and inactivation of the second allele by mutation, deletion, or hypermethylation (3, 4). *VHL* inactivation is the earliest event in tumor formation in the majority of sporadic ccRCC cases (5–9). In addition to *VHL* mutation, ccRCCs also frequently harbor different combinations of mutations in additional tumor suppressor genes that control diverse epigenetic functions, including *PBRM1*, *BAP1*, *SETD2*, and *KDM5C* (3, 4, 10).

An intriguing molecular link between *VHL* mutation as the main truncal ccRCC driver mutation and secondary mutations in the different ccRCC epigenetic tumor suppressor genes is that these proteins all normally control aspects of DNA repair and DNA damage signaling. In response to DNA damage, checkpoint kinase 2 phosphorylates the protein product of the *VHL* gene (pVHL), which promotes the formation of a complex involving pVHL, p53, ataxia telangiectasia mutated (ATM), and p300, promoting the activation of p53 by phosphorylation and acetylation, as well as preventing its ubiquitination and degradation, thereby acting as a positive regulator of p53 activity (11, 12). Suppressor of cytokine signaling 1 promotes K63 ubiquitination of pVHL in response to DNA damage, inducing the nuclear redistribution of pVHL, which has been shown to be necessary for efficient induction of DNA damage signaling and repair (13). *VHL*-mutant ccRCC cells exhibit decreased levels of expression of several proteins involved in homologous recombination repair and mismatch repair and show functional defects in these repair pathways (14). Nucleotide excision repair is also reduced in *VHL*-null ccRCC cell lines (15). These phenotypes are all rescued by reintroduction of wild-type pVHL (14, 15). Deletion of *Vhl* in mouse renal epithelial cells in vivo causes replication stress (16). In addition to their roles as regulators of chromatin structure in governing gene transcription, numerous chromatin-modifying proteins, including those that are frequently mutated in ccRCC, have been implicated in DNA repair and DNA replication fidelity. Polybromo 1 (PBRM1) functions to silence transcription around sites of DNA damage, thereby promoting DNA repair (17–19). This activity is abrogated by cancer-associated *PBRM1* mutations (17). BRCA1 associated protein 1 (BAP1) is required for DNA double-strand break repair by homologous recombination (20–22). SET domain containing 2 (SETD2) is important for homologous recombination repair of DNA double-strand breaks (23, 24), as well as DNA damage–induced signaling to ATM and p53 (25). Lysine demethylase 5C (KDM5C) is essential for proper DNA replication at early origins during S phase (26), and *KDM5C* mutation in ccRCC leads to uncontrolled expression of heterochromatic noncoding RNAs that cause increased genomic instability (27). Somatic and germline mutations in other genes that encode core components of DNA damage signaling and repair pathways, including *CHEK2*, *ATM*, *MSH6*, *MUTY*, *NBN*, *MSH2*, and poly(ADP-ribose) polymerase 1(*PARP1*), have been identified collectively in 19% of metastatic ccRCCs (28). Thus, in ccRCC there is a remarkable mutational convergence on genes that regulate DNA repair and DNA damage signaling.

Given the unique genetic underpinnings of the disease, ccRCC appears at least theoretically to be well suited to therapy using DNA-damaging chemotherapeutic agents. However, conventional chemotherapeutic regimens are not used for the treatment of metastatic ccRCC (2). Numerous phase I, II, and III clinical trials have tested a range of chemotherapeutic agents used alone or in combination with other chemotherapies, with immune-modulating cytokines, and with molecularly targeted therapies (reviewed in ref. 29). Although many agents were not successful, or were less successful than other available therapies, some of these trials nonetheless revealed some encouraging clinical responses, even in patients who were refractory to other agents and who had high metastatic burdens, demonstrating in principle that chemotherapeutic regimens might potentially be improved upon and might become more clinically useful in the future.

In this context, a new generation of pharmacological agents have recently been developed that inhibit the ATM, ATR, and DNA-dependent protein kinase (DNA-PK) DNA damage signaling kinases and thereby block normal DNA damage–induced signaling and repair processes (30–33). These classes of drugs have shown promising activities in preclinical and clinical studies in a range of tumor types. However, they have not been investigated in the context of ccRCC to our knowledge. In this study, we conducted detailed analyses of the response of ccRCC cell lines to conventional DNA-damaging chemotherapeutic agents as well as to ATM, ATR, and DNA-PK inhibitors (hereafter termed ATRi, ATMi, and DNA-PKi). We identified that ccRCC cells are sensitive to ATRi in cell culture and in xenografts. Therapeutic synergy between ATRi and several chemotherapeutic agents was observed across multiple ccRCC cell lines. We also show that combined ATRi plus cisplatin treatment has excellent therapeutic activity in xenograft and autochthonous ccRCC models. These studies identify a new class of molecular targets for ccRCC therapy.

## Results

### ccRCC cell lines and tumors exhibit high levels of endogenous DNA damage.

**Figure 1:**
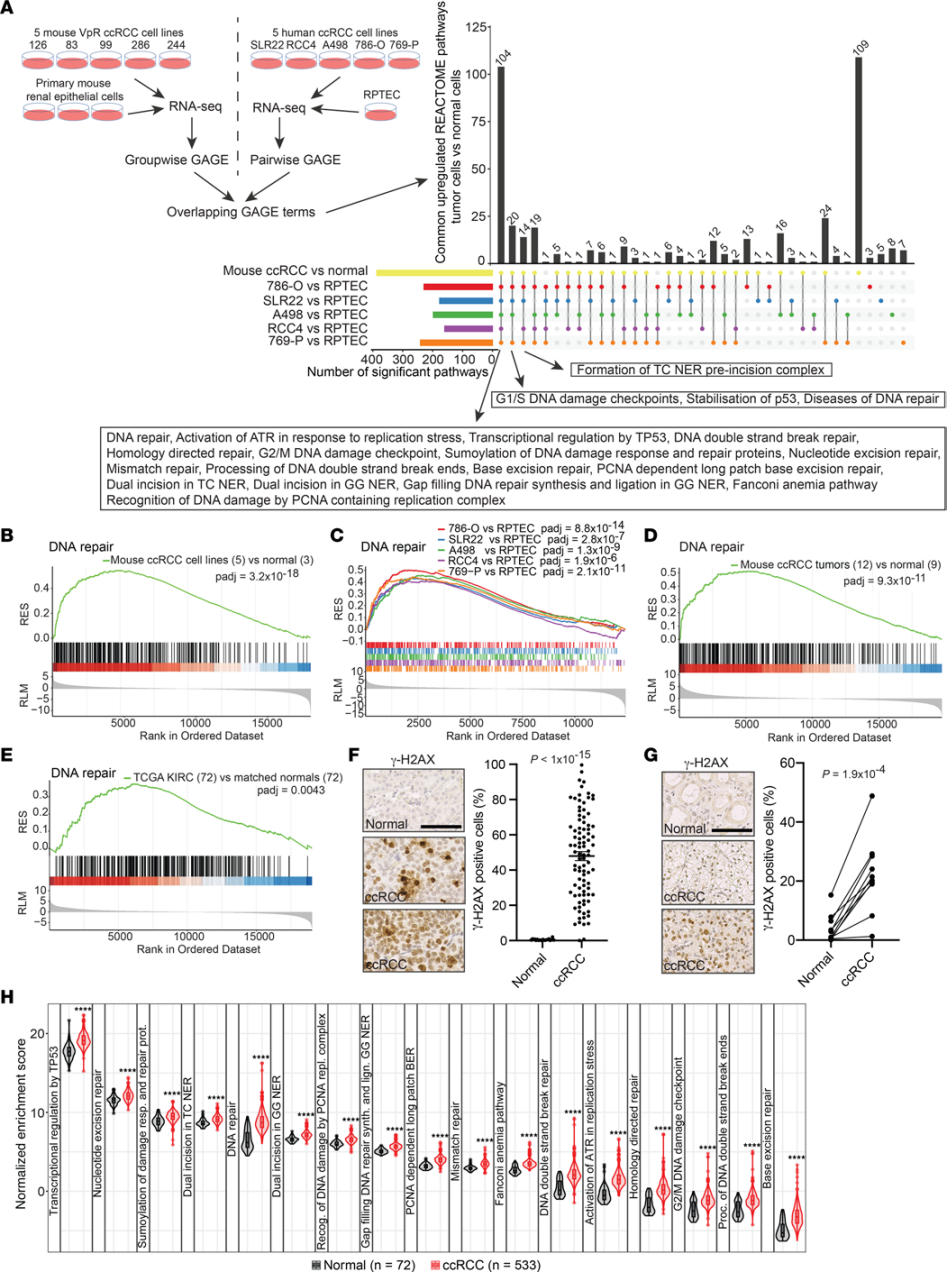
Endogenous DNA damage in ccRCC. (**A**) Experimental overview and summary of overlaps in enriched Reactome gene sets between comparisons of mouse ccRCC with normal primary cells and human ccRCC cell lines with RPTECs. UpSetR plot depicts the number of significantly enriched terms (Benjamini-Hochberg adj. *P* < 0.05) within each individual comparison (*x* axis) and of overlapping terms (*y* axis) between the indicated comparisons (dots). Box lists DNA damage–relevant terms that overlap between at least 5 individual comparisons. (**B**–**E**) Gene set enrichment plots for Reactome term “DNA repair” for mouse ccRCC cell line versus normal primary cells (**B**), human ccRCC cell lines versus RPTECs (**C**), mouse ccRCC tumors versus normal mouse renal cortex (**D**), and human ccRCC tumors (TCGA-KIRC) versus matched normal (**E**). Numbers in brackets represent number of samples. Colored lines show the running enrichment score (RES). Ranked list metric (RLM) is shown (bottom). *P* values were adjusted using Benjamini-Hochberg correction. (**F**) Immunohistochemical staining for γ-H2AX in normal mouse renal cortex and ccRCC in *Vhl/Trp53/Rb1* mutant mice and quantification of positively stained nuclei in normal (*n* = 10) and ccRCC (*n* = 92 tumors from 16 mice). Mean ± standard deviation (std. dev.). Two-sided *P* value calculated by Student’s unpaired *t* test with Welch’s correction. Scale bar = 50 μm. (**G**) Immunohistochemical staining for γ-H2AX in normal human renal cortex and ccRCC and quantification of positively stained nuclei in paired samples of normal and ccRCC (*n* = 10). Two-sided *P* value calculated by Student’s paired *t* test. Scale bar = 50 μm. (**H**) Distribution of TCGA-KIRC sample-specific normalized enrichment scores (NESs) for Reactome pathways. Violins show the kernel probability density of the NES deriving from ssGSEA; boxes indicate median and interquartile range (between 25th and 75th percentiles). Groups were compared using 2-sided Mann-Whitney *U* tests without multiple comparisons (****P < 0.0001). VpR, *Vhl/Trp53/Rb1* mutant cell lines.

To identify new biological pathways in ccRCC that could be therapeutically targetable, we conducted RNA-Seq of 5 independent ccRCC cell lines derived from the *Vhl/Trp53/Rb1*-deficient autochthonous mouse ccRCC model (34) and compared the RNA-Seq data with RNA-Seq data from 3 cultures of normal primary mouse renal epithelial cells from the same genetic background (Supplemental Data 1; supplemental material available online with this article; https://doi.org/10.1172/jci.insight.156087DS1). We also conducted RNA-Seq of 5 human ccRCC cell lines (786-O, 769-P, A498, SLR22, RCC4) and of primary renal proximal tubule epithelial cells (RPTECs) (Supplemental Data 2). Exome sequencing of the 786-O, A498, SLR22, and RCC4 human ccRCC cell lines, as well as analyses of mutations in 769-P cells published in the DepMap-CCLE database, revealed that all cell lines harbored *VHL* mutations as well as mutations in 1 or more of the *PBRM1*, *BAP1*, *SETD2*, or *KDM5C* genes or 1 of the DNA damage– or DNA repair–related genes *ATM*, *TP53*, *MSH6*, *PMS1*, or *POLE* (Supplemental Figure 1A and Supplemental Data 3). These cell lines therefore reflect the diversity of genetic mutations that arise in human ccRCC. We conducted generally applicable gene set enrichment (GAGE) analyses using the Reactome pathway database to define enriched gene expression signatures for the mouse tumor cell lines versus normal cells as well as for each human ccRCC cell line compared with RPTECs (Figure 1A and Supplemental Data 4). Analyses of the overlaps in Reactome signatures revealed that all tumor cell lines showed strong upregulation of signatures associated with many different aspects of DNA repair pathways, signaling of DNA damage, and transcriptional responses to DNA damage (Figure 1A). Examples of gene set enrichment plots for the term “DNA repair” are shown for mouse tumor cell lines versus primary renal epithelial cells (Figure 1B) and for the human ccRCC cell lines compared with RPTECs (Figure 1C). Similar GAGE analyses were conducted using RNA-Seq data from *Vhl/Trp53/Rb1*-deficient mouse tumors versus normal renal cortex (Figure 1D) and from matched normal and ccRCC tumor samples from The Cancer Genome Atlas Kidney Renal Clear Cell Carcinoma (TCGA-KIRC) database (Figure 1E). These analyses also revealed enrichment of DNA damage signatures, demonstrating that these gene expression profiles are also present in ccRCC tumors in vivo and are not an artifact of cell culture.

To corroborate the bioinformatic signatures of DNA damage, we stained normal kidneys from 10 non-Cre mice and 92 *Vhl/Trp53/Rb1*-deficient mouse ccRCCs from 16 mice with an antibody against the double-strand break DNA damage marker γ-H2AX and quantified the percentage of positively stained nuclei per tumor or region of normal cortex. Tumors exhibited varying frequencies of cells with DNA damage; only 2 tumors showed an absence of γ-H2AX staining while the remaining tumors exhibited positive frequencies in the range of 9%–100% (Figure 1F). We also stained 10 human renal biopsies that contained both normal tissue and ccRCC tumors. These analyses revealed that the human tumors exhibited higher levels of DNA damage than normal tissue, with tumor-to-tumor variation in the frequency of positively stained cells (Figure 1G).

To extend this observation to a larger set of tumors, we conducted single-sample gene set enrichment analyses (ssGSEAs) of 72 normal renal samples and of 533 ccRCC tumors from the TCGA-KIRC data set using previously identified Reactome DNA damage–related gene signatures (Supplemental Data 5). ssGSEA provides an enrichment score for each sample, which allows assessment of the relative strength of the gene expression signature in each individual normal or tumor sample. These single-sample analyses revealed that there were statistically significant differences between normal and ccRCC in all the DNA damage signatures, validating the results of the above-described analyses based on pooled tumors, as well as revealed a large variation in the degree of enrichment of these signatures among ccRCC tumors, ranging from being not enriched in comparison to normal kidney to being highly enriched (Figure 1H). We next investigated whether the DNA repair signature is specific to ccRCC or whether this signature is present in other tumor types by conducting ssGSEAs of 21 additional sets of TCGA RNA-Seq data from tumors and corresponding normal tissues (Supplemental Figure 2A). In all but 3 tumor types, the DNA repair signature was enriched in tumors versus normal, consistent with the known high rates of DNA damage in most cancers, highlighting that ccRCC is not a unique outlier that is resistant to chemotherapy due to the absence of DNA damage in this tumor type. Correlation of the strength of the DNA repair signature with mRNA expression of the proliferation markers *MKI67* (Supplemental Figure 2B) or *PCNA* (Supplemental Figure 2C), or with enrichment of an mRNA signature of proliferation-associated genes (Supplemental Figure 2D), revealed a positive correlation between proliferation and the enrichment of the DNA repair signature, consistent with proliferation-associated DNA damage as being one contributor to the enrichment of mRNA signatures of DNA damage in ccRCC.

Given their roles in DNA damage-related processes, we next investigated whether the presence of mutations in *VHL*, *PBRM1*, *BAP1*, *SETD2*, *KDM5C*, or *TP53* correlated with the enrichment of any of the different DNA damage signatures by comparing enrichment scores between normal kidney and ccRCC with or without a mutation in each of the genes individually (Supplemental Figure 3). Indeed, tumors with mutations in *PBRM1*, *BAP1*, *SETD2*, or *TP53* showed higher enrichment scores for specific DNA damage-related signatures, and these signatures were only partially overlapping for each gene. *PBRM1* mutations were associated with higher signatures for DNA double-strand break repair and G_2_/M DNA damage checkpoint; *BAP1* mutations with mismatch repair, Fanconi anemia pathway, ATR activation, and homology directed repair; *SETD2* mutations with several different nucleotide excision repair signatures, 2 base excision repair signatures, and general terms — processing of DNA double-strand break ends, recognition of DNA damage by PCNA-containing replication complex, DNA repair, and transcriptional regulation by TP53; and finally *TP53* mutations with high enrichment scores for nucleotide excision repair and ATR activation. Keeping in mind that clones of tumor cells often harbor multiple mutations in these genes (7–9), it is likely that this analysis underestimates the contributions of each mutation individually, and potentially cooperatively, in causing DNA damage in ccRCC. These analyses nonetheless support the idea that DNA damage in ccRCC can arise as a consequence of mutations in several tumor suppressor genes.

In summary, we conclude that endogenous DNA damage is a frequent feature of ccRCC tumors and that there is variability among tumors in terms of the frequency of cells with DNA damage.

### Normal cellular responses to DNA damage in ccRCC cell lines.

**Figure 2:**
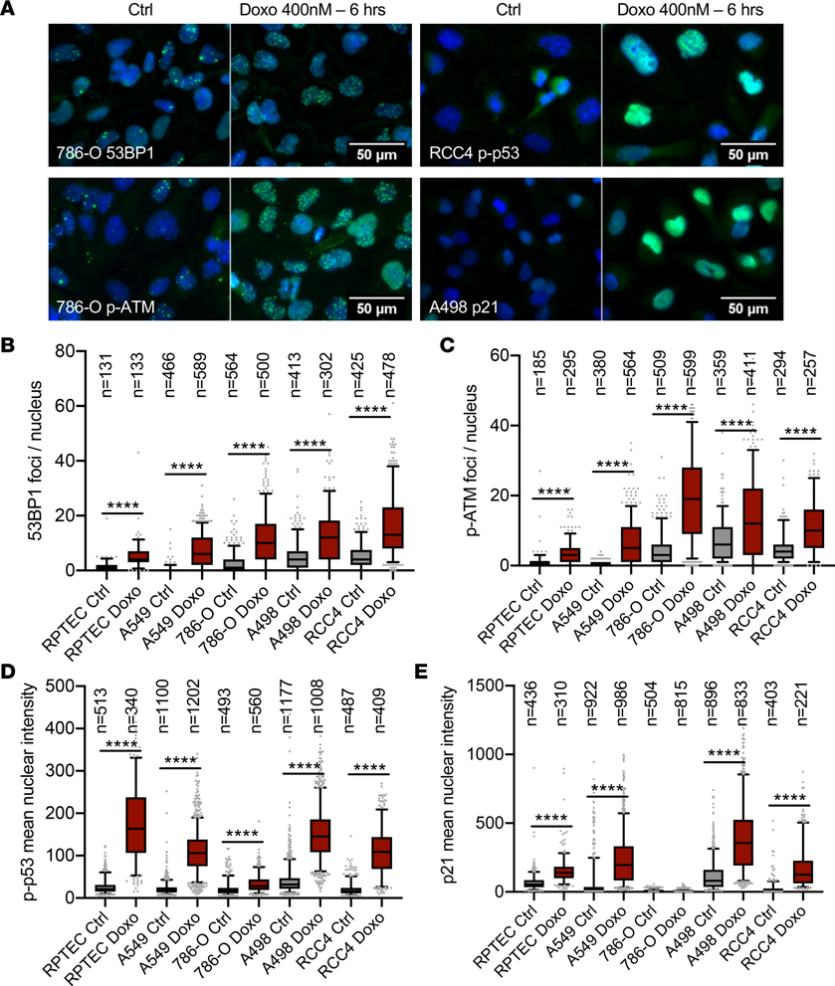
Normal DNA damage induction and sensing in ccRCC cells. (**A**) Examples of immunofluorescence stainings for 53BP1, p-ATM, p-p53, and p21 in 786-O, RCC4, and A498 control or doxorubicin-treated ccRCC cell lines. (**B**) Quantification of nuclear 53BP1 foci. (**C**) Quantification of nuclear p-ATM foci. (**D**) Quantification of mean nuclear p-p53 intensity. (**E**) Quantification of mean nuclear p21 intensity. Box plots show the quartiles, whiskers depict 5th through 95th percentiles, and extreme values to the maximum and minimum are shown by gray dots. The number of nuclei analyzed per condition are shown (*n*), and statistical differences between groups were assessed with the nonparametric Mann-Whitney test (****P < 0.0001).

To investigate whether the clinical chemotherapy resistance of ccRCC is due to the lack of an intrinsic response, or relative resistance, to DNA damage, we compared DNA damage responses between 3 human ccRCC cell lines (786-O, RCC4, and A498), normal RPTECs, and lung adenocarcinoma A549 cells, which represent a positive control cell line that we have previously identified as having low basal levels of DNA damage and strong and reproducible DNA damage responses (our unpublished observations). Cells were treated for 6 hours with a low dose (400 nM) of the DNA double-strand break–inducing topoisomerase II inhibitor doxorubicin. We used quantitative immunofluorescence-based analyses to gain single-cell resolution of readouts of physical DNA damage (53BP1 focus formation) and activation of DNA damage response signaling by staining for nuclear phosphorylated S428-ATM (hereafter p-ATM), phosphorylated S15-p53 (hereafter p-p53), and p21 (Figure 2A). Untreated ccRCC cell lines displayed increased numbers of 53BP1 foci and p-ATM nuclear accumulation compared with normal RPTECs and with A549 cells (Figure 2, B and C), indicative of higher levels of basal DNA damage. All cell lines responded to doxorubicin by increasing these DNA damage markers (Figure 2, B and C). Downstream phosphorylation of p53 and induction of p21 expression were induced in A549 cells, RPTECs, RCC4 cells, and A498 cells, but were absent in 786-O cells (Figure 2, D and E), which is consistent with the fact that these latter cells harbor mutations in *TP53*. DNA damage signaling therefore appears to be induced normally, at least in p53 wild-type ccRCC cells.

Given the numerous reported connections between pVHL and DNA damage repair processes, it is believed that mutation of *VHL* may alter the sensitivity of ccRCC cells to DNA-damaging agents. To test this idea, we next investigated whether retroviral reintroduction of pVHL expression in *VHL*-null ccRCC cell lines might alter cellular responses to doxorubicin. We first confirmed that pVHL reintroduction reduced expression of HIF-1α and HIF-1α or HIF-2α target proteins GLUT1, CA9, NDRG1, and Cyclin D1 as expected (Supplemental Figure 1B; see complete unedited blots in the supplemental material), confirming functional rescue of *VHL* deficiency. To discriminate between DNA damage induction and DNA damage repair, cells were analyzed directly following 6 hours of doxorubicin treatment and following an additional 24-hour recovery phase in the absence of doxorubicin (Supplemental Figure 4, A–D). In general, pVHL rescue did not affect the induction of DNA damage (53BP1 foci) or the induction of DNA damage signaling (p-ATM, p-p53, and p21). RCC4 cells more efficiently repaired DNA and limited DNA damage signaling after 24 hours than 786-O and A498 cells.

**Figure 3:**
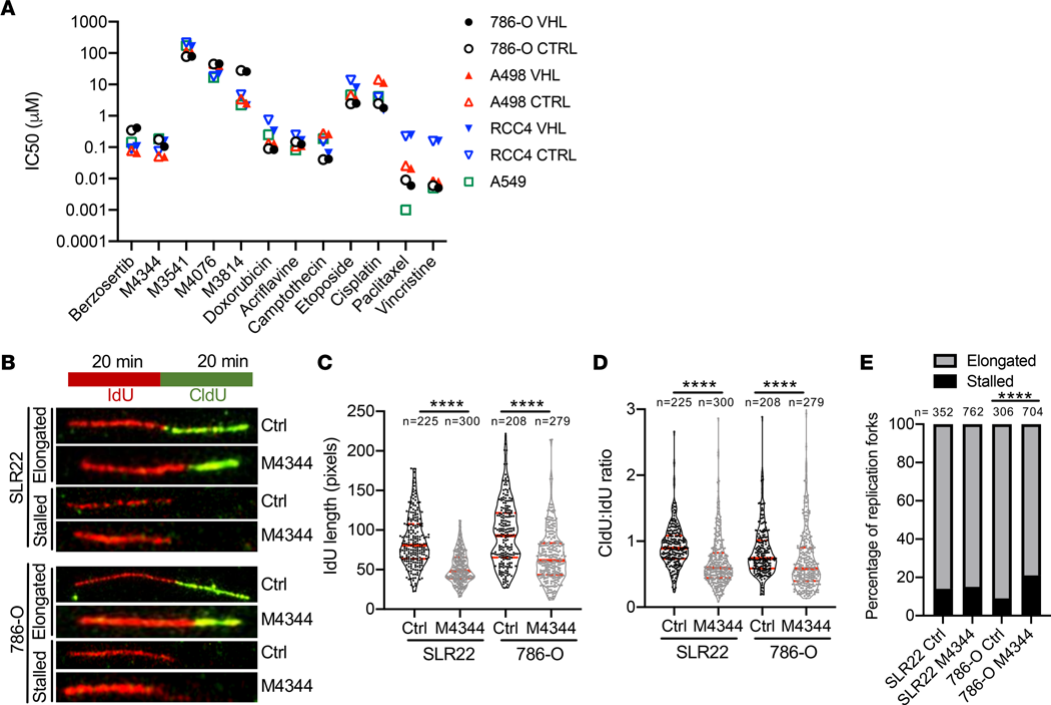
M4344 induces replication stress in ccRCC cells. (**A**) IC_50_ values based on 72-hour cell viability assays of the indicated cell lines infected with empty vector (CTRL) or pVHL-expressing vector (VHL) for the indicated drugs. (**B**) Examples of elongated and stalled DNA fibers in control or M4344-treated SLR22 and 786-O cells. (**C**) Quantification of the length of IdU-labeled fibers. (**D**) Quantification of the ratio of the lengths of CIdU to IdU in DNA replication fibers. Violin plots in **C** and **D** depict individual values, solid red lines show median, dotted red lines depict upper and lower quartiles, the number of analyzed fibers (*n*) is shown, and statistical differences between groups were assessed with the nonparametric Mann-Whitney test (****P < 0.0001). (**E**) Percentage of stalled and elongated replication forks. The number of analyzed fibers (*n*) is shown, and statistical differences between groups were assessed with Fisher’s exact test (****P < 0.0001).

One consequence of failure to repair DNA damage and continued cell cycle progression is the formation of micronuclei following mitosis (35–38). The presence of micronuclei therefore serves as an indirect readout of longer term responses to DNA damage. Using an automated segmentation algorithm (Supplemental Figure 5A), we quantified the average number of micronuclei per nucleus over 4 days following treatment for 6 hours with doxorubicin in RCC4 and 786-O cells infected with vector control or expressing pVHL. These studies revealed that micronuclei were formed in both cell lines and that there were no statistically different changes in the time course of appearance of micronuclei (Supplemental Figure 5, B and C) in the parental or pVHL–rescued cell lines. To complement these molecular DNA damage analyses, we performed long-term cytotoxicity assays in RCC4, 786-O, and A498 vector control and pVHL-rescued cell lines in the presence or absence of doxorubicin for 48 hours followed by 6 days of recovery prior to analysis (Supplemental Figure 5, D–F). Doxorubicin reduced survival in a dose-dependent manner in all cell lines. pVHL reintroduction slightly reduced survival following doxorubicin treatment in RCC4 and A498 but not 786-O cells. We next extracted data from the Genomics of Drug Sensitivity in Cancer Project (https://www.cancerrxgene.org). This database contains IC_50_ survival data for around 1,000 human cancer cell lines (including 50 renal carcinoma lines) and around 400 different drugs. We compared IC_50_ values of renal carcinoma cell lines with cell lines from other human tumor types for the DNA-damaging drugs camptothecin, cisplatin, doxorubicin, paclitaxel, olaparib, and etoposide (Supplemental Figure 5, G–L). These analyses revealed no differences in the range of sensitivities of renal carcinoma cell lines to these agents compared to cell lines derived from other tumor types in which these chemotherapeutic drugs are used therapeutically. We extended these analyses by determining IC_50_ values of a series of standard chemotherapeutic agents as well as of several specific kinase inhibitors of the effectors of DNA damage responses, namely 2 ATRi (M4344 and M6620, formerly VX-970, now termed berzosertib), 2 ATMi (M3541 and M4076), and 1 DNA-PK inhibitor (M3814, now termed peposertib) in 786-O, A498, and RCC4 vector control and pVHL-rescued cells (Figure 3A). pVHL reintroduction did not alter sensitivity to any of these agents. In summary, we conclude that *VHL* mutation does not render ccRCC cells intrinsically resistant to the molecular and cellular consequences of DNA-damaging agents.

### ATRi induce replication stress in ccRCC cells.

Recent studies identified replication stress gene expression signatures as predictive of responses to ATRi (39, 40). Given that we identified numerous signatures of DNA damage, including the Reactome term “activation of ATR,” in response to replication stress as features of mouse and human ccRCC, it is notable that the 2 ATRi showed IC_50_ values that were 100- to 1,000-fold lower than the ATMi and DNA-PKi in ccRCC cell lines (Figure 3A). We therefore sought to further investigate the mechanisms through which ATRi result in potent toxicity in ccRCC cells. ATR predominantly signals single-stranded DNA breaks, which typically occur in S phase due to stalled replication forks (32). We first hypothesized that enhanced replication stress may contribute to the observed effects of ATRi in ccRCC cells, as has been observed in other cancer cell lines treated with ATRi (39). To test this idea, we first devised a flow cytometry workflow that allows the quantification of the extent of DNA replication completion within a population of cells (Supplemental Figure 6A). First, S phase cells were labeled by a pulse of EdU (30 minutes) followed by a recovery phase to allow DNA replication to progress. Next, cells were fixed and stained for EdU and DNA content (DAPI). By analyzing the average DNA content of these cells at the start and end of the recovery period, it is possible to quantitatively assess the extent to which the population of S phase cells has replicated their DNA. Since the cells in the culture are asynchronously progressing through the cell cycle, S phase cells will on average be halfway through DNA replication, reflected by an average DAPI intensity that is halfway between the DAPI intensity of G_0_/G_1_ and G_2_/M cells. Intuitively, if the average DNA content at the time point of analysis is higher than this value, then replication has progressed during the recovery phase; if the average DNA content is unchanged or only moderately increased, then replication has been blocked. An important additional point to include in the quantification of average DNA intensity is that some EdU-positive cells will complete S phase and divide during the recovery period. These cells appear in the flow cytometry plots as a population of EdU-positive G_0_/G_1_ cells. Therefore, only half the number of cells in this population are counted in the subsequent analyses, and these cells are counted at a DNA intensity of 4n, to reflect full completion of S phase. For each comparison, it is therefore possible to determine the fraction of DNA replication completion, with a value of 0.5 representing complete progression of the initial S phase population through S phase. An example of how this assay can be used to infer effects on DNA replication stress is the replication stress–inducing drug hydroxyurea that was added during the 7-hour recovery period (last flow cytometry plot in Supplemental Figure 6A). The DNA intensity profile of the population of EdU-positive, hydroxyurea-treated cells appeared similar to the sample of cells taken immediately following the EdU pulse. It also clearly lacked the shift in DNA intensity to the G_2_/M and G_0_/G_1_ populations that was seen in the control cells that recovered for 7 hours in the absence of drug.

Applying this method, we first compared the relative rates of S phase progression of ccRCC cell lines to other cancer cell lines, but did not observe any consistent differences, arguing that under untreated conditions ccRCC cell lines do not experience higher levels of replicative stress than other cancer cell lines that would impair S phase progression (Supplemental Figure 6B). Next, we tested replication stress in 786-O following treatment with M4344. Hydroxyurea treatment served as a positive control for replication stress (Supplemental Figure 6C). When cells were just treated during recovery phase (Supplemental Figure 6C), M4344 at high doses (2 μM) induced S phase arrest, and when cells were additionally pretreated for 24 hours, S phase arrest occurred for M4344 at concentrations of 200 nM and above (Supplemental Figure 6D). These observations suggest that M4344 slows progression through S phase at IC_50_-relevant doses. These data are consistent with the idea that enhanced levels of replication stress, or failure to resolve DNA damage in S phase, may contribute to the cytostatic effects of ATRi. To directly test this idea, we treated SLR22 and 786-O cells for 24 hours with M4344 and then conducted DNA fiber assays by labeling replicating DNA strands for 20 minutes with IdU, followed by 20 minutes of labelling with CIdU, all in the continued presence of M4344. Replicating DNA strands were identified as contiguous regions of IdU (red) and CIdU (green) labeling (Figure 3B). Quantification of DNA fiber lengths revealed that M4344 reduced the average length of IdU fibers (Figure 3C) as well as reduced the ratio of the length of CIdU- to IdU-labeled fibers (Figure 3D) in both cell lines. These results indicate a slower rate of DNA synthesis (or increased rate of replication stalling), likely due to ongoing replication stress that manifests stochastically during the first or second period of labeling for any given replicating DNA fiber. Quantification of the frequency of DNA fibers that were only labeled with IdU and not with CIdU (Figure 3B), indicative of replication forks that stopped during the period of the IdU labeling but did not resume replication within the 20-minute period of the CIdU incubation, revealed that M4344 increased replication fork stalling in 786-O cells, but not in SLR22 cells, which showed a higher basal level of stalled forks (Figure 3E). These findings are in agreement with the conclusions of our flow cytometry experiments of M4344 causing slower S phase progression in ccRCC cells.

### ATRi treatment induces DNA damage accumulation.

**Figure 4:**
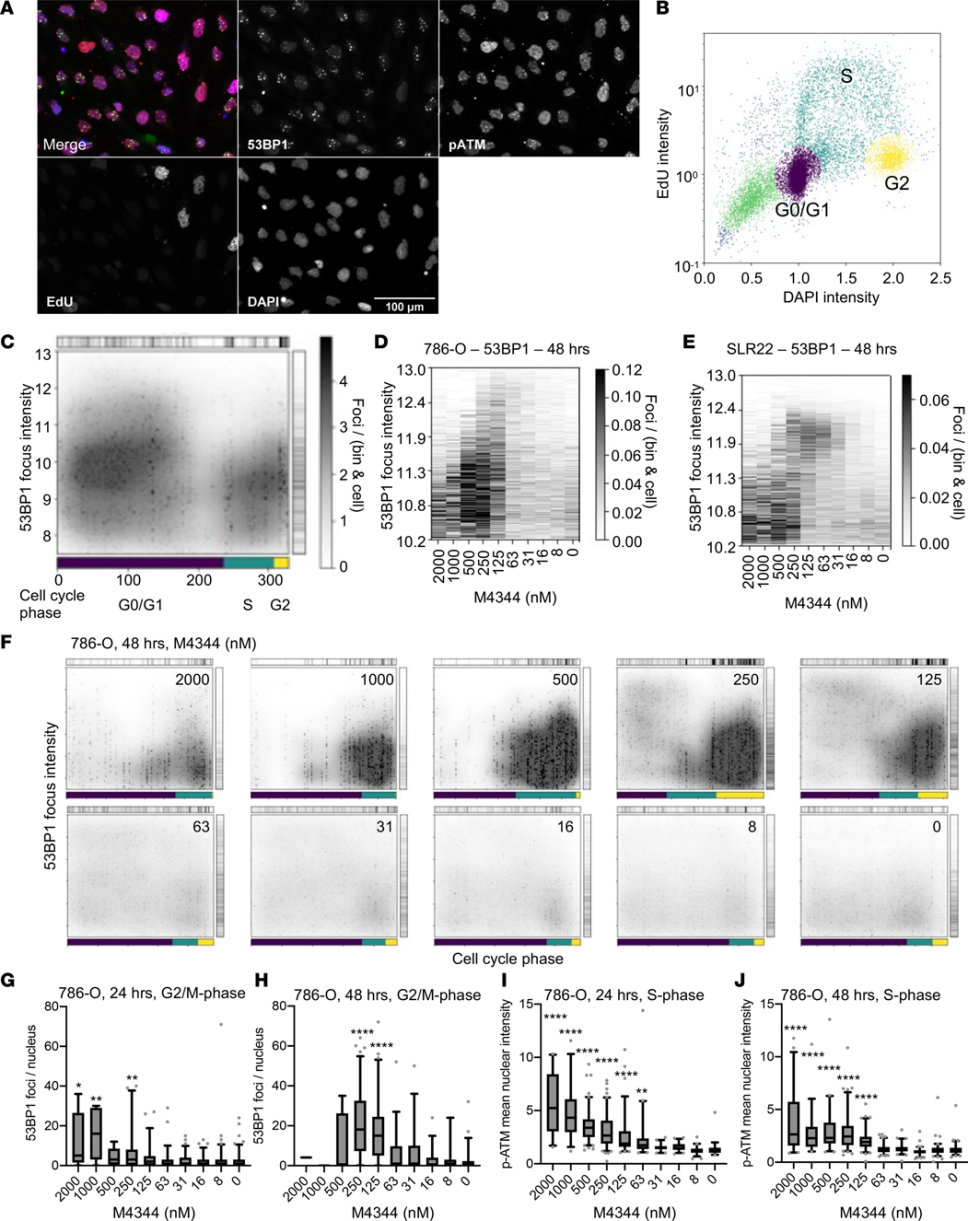
Characterization of DNA damage induced by M4344. (**A**) Examples of immunofluorescence stainings for 53BP1, p-ATM, EdU, and DAPI and merge of all signals. (**B**) Cell cycle phase resolution based on quantitative microscopy analysis of DAPI intensity and EdU staining. (**C**) Example of 1D (right bar) and 2D (cell cycle phase resolved) analysis of the distribution of 53BP1 focus intensities. (**D** and **E**) One-dimensional analyses of 53BP1 focus intensities in 786-O (**D**) and SLR22 (**E**) cells treated for 48 hours with the indicated concentrations of M4344. (**F**) Two-dimensional cell cycle resolution of 53BP1 focus intensities in 786-O cells treated for 48 hours with M4344. (**G** and **H**) Quantification of 53BP1 focus intensities in G_2_/M phase 786-O cells treated for 24 hours (**G**) or 48 hours (**H**). (**I** and **J**) Quantification of p-ATM mean nuclear intensities in S phase 786-O cells treated for 24 hours (**I**) or 48 hours (**J**). Box plots show the quartiles, whiskers depict 5th through 95th percentiles, and extreme values to the maximum and minimum are shown by gray dots. Statistical differences between M4344-treated versus control groups were assessed with the nonparametric Kruskal-Wallis test with Dunn’s correction for multiple comparisons (*P < 0.05, **P < 0.01, ****P < 0.0001). Sample sizes range from 9 to 114 nuclei per condition.

To more precisely investigate whether M4344-induced replication stress results in DNA damage, we developed a multiparametric immunofluorescence microscopy assay. Given the complex interplay between cell cycle phase and DNA damage detection, signaling, and repair (41), we integrated EdU and DAPI quantifications to permit cell cycle analysis combined with analyses of the DNA damage markers 53BP1 and p-ATM and detection of micronuclei. To this end, we applied a multiplex imaging technique that allows cyclic staining and image acquisition within the same samples (42), providing single-cell resolution of multiple parameters in several hundreds of individual cells in each experimental condition (Figure 4A). Quantitative image analyses allowed the assignment of cell cycle phase based on EdU staining and DAPI intensity, analogously to a flow cytometry analysis (Figure 4B), and analysis of 53BP1 permitted cell cycle phase–resolved quantification of DNA damage focus numbers and intensities. DNA damage foci are challenging to analyze, as different cellular states may produce foci of different characteristics (43). The intensity of the focus provides information about the extent and age of DNA damage at that site, with larger, brighter foci representing older or persistent DNA damage lesions (43). Hence for 53BP1 focus analysis, we developed 1- and 2-dimensional (1D and 2D) histogram visualizations to reflect the distribution of focus intensities with or without resolution of cell cycle status (Figure 4C). For the 1D analyses, foci were “binned” based on 53BP1 intensities, and the gray scale depicts the number of foci within that intensity bin, averaged across the cellular population. In 2D representations (Figure 4C), cells were arranged on the *x* axis based on DAPI content and ascribed a cell cycle phase as illustrated in Figure 4B. Measuring total nuclear levels of p-ATM gave additional information about ongoing DNA damage response signaling, which may or may not correlate with enhanced DNA damage focus numbers. We also quantified micronuclei to gain cell cycle–resolved information about enhanced rates of mitotic aberrations.

We treated 786-O and SLR22 cells with a dilution series of M4344 for 24 or 48 hours. Analyses of the 1D distribution of 53BP1 focus intensities after 48 hours of treatment revealed that M4344 induced DNA damage, with dose-dependent effects on the intensities of foci that formed (Figure 4, D and E). Given these observations, we next resolved the DNA damage with respect to cell cycle phase (Figure 4F). In 786-O cells, 48-hour treatment with moderate doses of M4344 (125 nM and 250 nM) induced moderate accumulation of DNA damage in S phase cells, a very strong DNA damage accumulation in G_2_/M, and moderate accumulation of more intense 53BP1-marked DNA damage foci in G_0_/G_1_ cells. This observation is consistent with cells accumulating damage in S phase, but still being able to exit S phase with unresolved replication stress, either arresting in G_2_/M or progressing through mitosis into G_0_/G_1_ with unresolved DNA damage. This conclusion is supported by the increased G_2_/M fraction and decreased G_0_/G_1_ fraction of cells in the analyses (Figure 4F). Higher M4344 doses induced strong DNA damage accumulation in S phase cells and an almost complete absence of G_2_/M cells, consistent with stronger DNA damage inducing an almost complete S phase cell cycle arrest. A similar pattern was observed in SLR22 cells (Supplemental Figure 7A), albeit with an apparently shifted dose-response relationship, where low doses (up to 63 nM) induced accumulation of DNA damage predominantly in G_0_/G_1_ cells, while moderate doses (125–250 nM) induced damage in G_2_/M and G_0_/G_1_ cells and higher doses caused strong damage in S phase and G_2_/M phase, accompanied by cell cycle arrest in these phases.

Comparisons of 24- and 48-hour drug treatments additionally revealed features of the kinetics of DNA damage and DNA damage signaling. Higher concentrations of M4344 led to DNA damage in G_2_/M phase cells within 24 hours, and lower doses induced damage within 48 hours in both cell lines (Figure 4, G and H, and Supplemental Figure 7, B and C). DNA damage signaling, as measured by nuclear accumulation of p-ATM, was strongly induced by M4344 in S phase cells after 24 and 48 hours in both cell lines (Figure 4, I and J, and Supplemental Figure 7, D and E). Western blotting analyses of 786-O, SL22, RCC4, and A498 cells treated for 24 or 48 hours with M4344 corroborated the conclusions of the microscopy experiments and showed that M4344 treatment increased the abundance of the DNA damage marker γ-H2AX as well as increased p-ATM levels, despite reduction in overall ATM levels in 3 of 4 cell lines (Supplemental Figure 8). Taken together, these observations suggest that M4344 induces the rapid accumulation of DNA damage in S phase cells. The cell cycle phenotype is dependent on the concentration of the drug, but in general, 786-O and SLR22 cells can show arrest in S phase or progress to the subsequent G_2_/M and G_0_/G_1_ phases with damaged DNA. This conclusion of cell cycle progression in the presence of DNA damage is further supported by analyses of micronuclei. Across all drug treatments in both 786-O cells (Supplemental Figure 9A) and SLR22 cells (Supplemental Figure 7F), micronuclei were found to be more closely associated with nuclei that were in the G_0_/G_1_ phase than those that were in S or G_2_/M phases. Furthermore, the G_0_/G_1_ nuclei that were close to micronuclei showed higher intensities of 53BP1 foci than those that were closer to S or G_2_ nuclei, suggesting that the G_0_/G_1_ foci were carried over from prior S phase DNA damage rather than being newly formed during G_1_. These observations are consistent with the idea that cells that progress through G_2_/M with higher levels of unresolved DNA damage are more likely to experience mitotic abnormalities that generate micronuclei when the cell exits mitosis and enters G_0_/G_1_ phase. A similar conclusion can also be drawn from dose-response analyses of 53BP1 foci in G_0_/G_1_ cells and micronuclei per cell (Supplemental Figure 7, G and H, and Supplemental Figure 9, B and C), which showed a strong statistical correlation between the extent of DNA damage in G_0_/G_1_ cells and the formation of micronuclei (Pearson’s correlation coefficients of 0.90 and 0.92 for 786-O and SLR22, respectively). These observations are consistent with the relationship between replication stress, unresolved DNA damage, and mitotic defects that lead to the formation of micronuclei (35, 38). Hence, although abrogation of the normal replication stress repair response was the primary mode of drug-induced damage in these 2 ccRCC cell lines, there was also a subsequent increase in persistent DNA damage in G_2_/M phases that led to mitotic defects, the formation of micronuclei, and the presence of DNA damage foci in G_0_/G_1_ cells. All of these different consequences likely contribute to the antiproliferative and cytotoxic activity of ATRi.

### ATRi inhibit ccRCC xenograft tumor growth.

**Figure 5:**
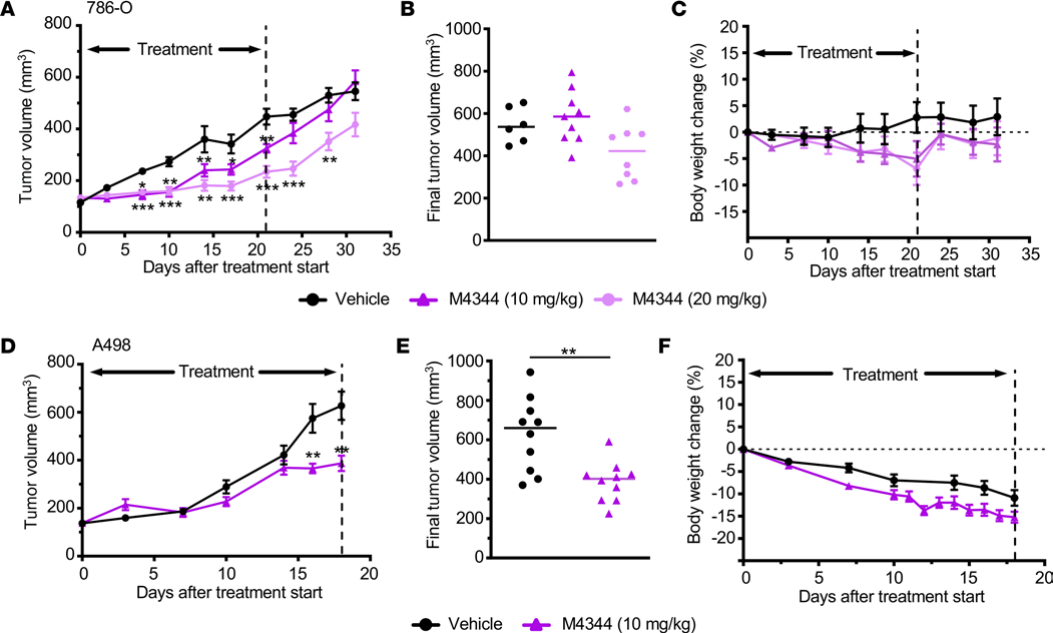
Therapeutic activity of M4344 in ccRCC xenografts. (**A**–**C**) Tumor growth curves (**A**) (mean ± SEM, *n* = 6 vehicle and *n* = 10 M4344), final tumor volumes (**B**), and body weight changes (**C**) in 786-O xenografts treated with vehicle or with 10 mg/kg or 20 mg/kg M4344 daily by gavage. The time point of the last treatment is indicated. (**D**–**F**) Tumor growth curves (**D**) (mean ± SEM, *n* = 10 vehicle and *n* = 10 M4344), final tumor volumes (**E**), and body weight changes (**F**) in A498 xenografts treated with vehicle or with 10 mg/kg M4344 daily by gavage. The time point of the last treatment is indicated. Two-sided *P* values comparing drug treatment with vehicle control were calculated by Student’s *t* test, *P < 0.05, **P < 0.01, ***P < 0.001.

To validate the antiproliferative and cytotoxic properties of ATRi in vivo, Rag2 mice bearing subcutaneous 786-O and A498 xenografts were treated once daily with 10 mg/kg or 20 mg/kg M4344 or vehicle. ATRi significantly decreased tumor growth rates and final tumor sizes in both 786-O and A498 models (Figure 5, A, B, D, and E). Compared with vehicle-treated animals, treatment with M4344 induced moderate weight loss in 786-O and A498 tumor–bearing mice (Figure 5, C and F). A498 tumor growth, associated with cachexia and weight loss, also occurred in vehicle-treated animals. These findings establish that ATRi slow tumor growth of ccRCC xenografts.

### Identification of drug treatment synergies in ccRCC cells.

**Figure 6:**
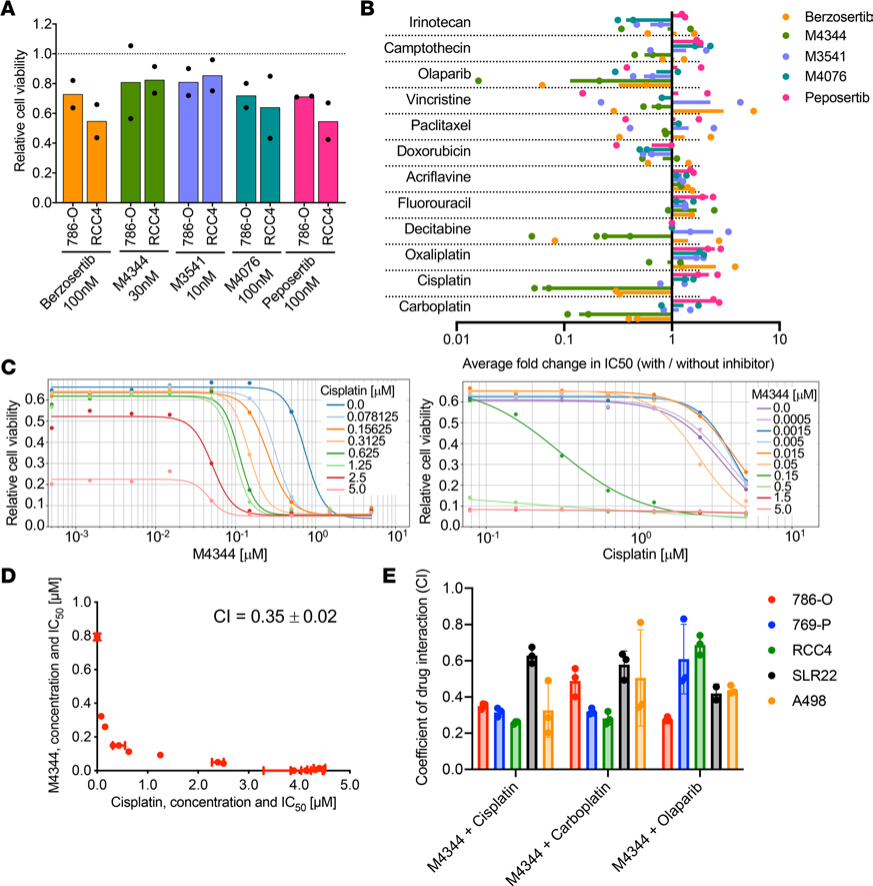
Chemotherapeutic synergies in ccRCC cells. (**A**) Effects of the indicated doses of ATRi, ATMi, and DNA-PKi as single agents on cell viabilities of the listed cell lines. Mean values obtained in 2 independent (represented by dots) drug screens are shown. (**B**) Change in IC_50_ values (bars show mean values of screens using 786-O and RCC4, represented by dots) for each agent in the presence compared with absence of ATMi, ATRi, and DNA-PKi. (**C**) Representative drug interaction dose-response curves of M4344 and cisplatin in 786-O cells. (**D**) Isobologram visualization of drug synergy analysis of M4344 and cisplatin in 786-O cells. Data depict mean ± std. dev. of 3 independent experiments, and the calculated coefficient of drug interaction (CI) is shown. (**E**) CI values for the indicated combinations of drugs in the indicated ccRCC cell lines. Data depict mean ± std. dev. of 3 independent experiments.

The above-described results provided evidence that targeting DNA damage signaling could be therapeutically beneficial in ccRCC. To attempt to develop more potent therapies, we next sought to identify combination treatments involving ATRi, ATMi, and DNA-PKi. A desired feature of a combination therapy is that it exhibits synergy, meaning that the therapeutic effect of the combination treatment is greater than the additive effects of the individual treatments, and that synergy is observed across a range of concentrations of the 2 drugs, a potentially important consideration in the pharmacological context of different half-lives of drugs in vivo. In a cell culture–based screen we used single concentrations of each of the ATR, ATM, and DNA-PK inhibitor drugs that caused approximately 20%–45% loss of viability in the ccRCC cells after 72 hours of treatment (Figure 6A). These concentrations were used as sensitizing doses in a screen of 12 DNA-damaging drugs that represent all major classes of chemotherapeutic agents, including topoisomerase inhibitors (irinotecan, doxorubicin, camptothecin), an intercalating agent (acriflavine), base analogs (fluorouracil, decitabine), adduct-forming agents (oxaliplatin, cisplatin, carboplatin), mitotic blockers (vincristine, paclitaxel), and a PARP inhibitor (olaparib). We conducted 72-hour survival assays in 96-well plate format with a chemotherapeutic drug dilution series in the presence or absence of the sensitizing dose of ATMi, ATRi, or DNA-PKi to determine IC_50_ values (Figure 6B). Most combination treatments did not show relevant alterations in relative IC_50_ values with or without ATMi, ATRi or DNA-PKi, which served as an internal control for the validity of the assay. We identified combinations of ATMi + irinotecan, ATRi + olaparib, ATRi + decitabine, ATRi + cisplatin, and ATRi + carboplatin as showing strong synergy. Impressively, M4344 showed synergies leading to 7- and 17-fold enhancements in IC_50_ values relative to single chemotherapeutic treatments. In a second series of experiments we conducted drug cooperativity assays to assess whether these identified combinations indeed showed synergies across a range of doses and whether these synergistic cytotoxic effects were observed in multiple ccRCC cell lines. Combination concentration-response screens were conducted for each cell line and drug combination. This allowed the determination of IC_50_ values of 1 drug at a series of fixed concentrations of the other drug and vice versa. Synergistic interactions were visualized by the shift in IC_50_ curves of 1 drug at increasing concentrations of the other (Figure 6C). The method of isoboles (44) (Figure 6D) was used to quantify these effects by calculating the CI, where CI values of less than 1 indicate synergistic interactions. The results of these CI assays are summarized in Figure 6E and demonstrate that M4344 showed synergy with cisplatin, carboplatin, and olaparib in each of the 786-O, 769-P, RCC4, SLR22, and A498 ccRCC cell lines. Similar synergies between ATR inhibitors and chemotherapeutic agents have been identified in other cancer cell types (39, 45). These studies identified a set of combinations to test in preclinical in vivo studies with ccRCC tumor models.

### ATRi plus cisplatin potently inhibits ccRCC growth in vivo.

**Figure 7:**
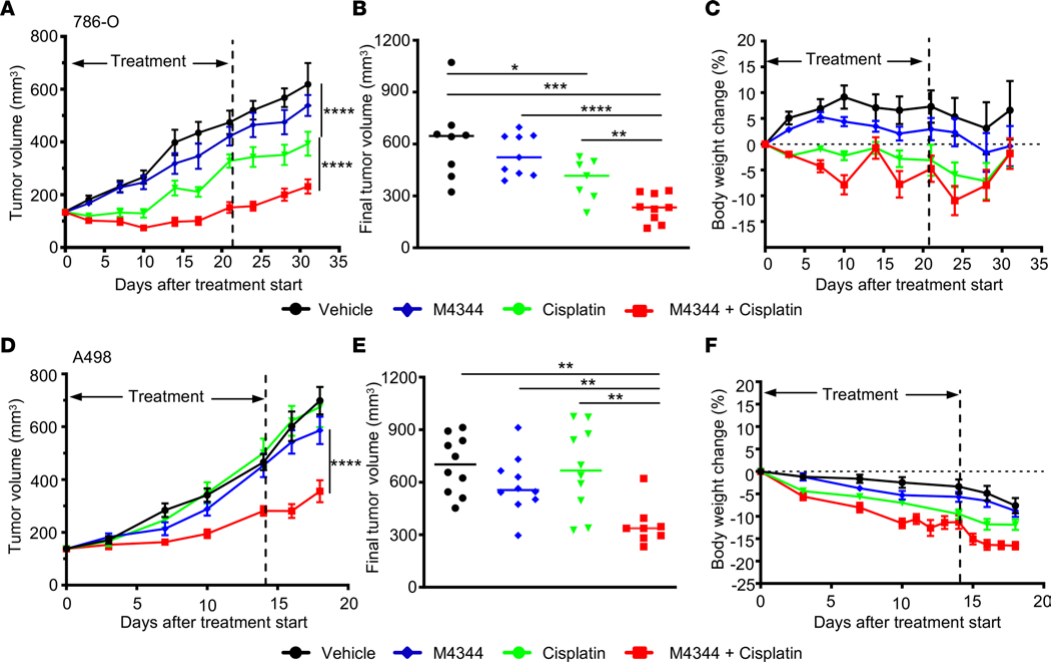
In vivo therapeutic efficacy of M4344 plus cisplatin in ccRCC xenografts. (**A**–**C**) Tumor growth curves (**A**), final tumor volumes (**B**), and body weight changes (**C**) in 786-O xenografts treated once weekly for 4 weeks with vehicle, with cisplatin (2.5 mg/kg), with M4344 (10 mg/kg), or with cisplatin (2.5 mg/kg) followed by M4344 (10 mg/kg) 18 hours later (mean ± SEM, *n* at treatment start = 10). The time point of the last treatment is indicated. (**D**–**F**) Tumor growth curves (**D**), final tumor volumes (**E**), and body weight changes (**F**) in A498 xenografts treated once weekly for 3 weeks with vehicle, with cisplatin (2.0 mg/kg), with M4344 (10 mg/kg), or with cisplatin (2.0 mg/kg) followed by M4344 (10 mg/kg) 18 hours later (mean ± SEM, *n* at treatment start = 10). The time point of the last treatment is indicated. Statistical analyses in **A** and **D** involved repeated measures ANOVA followed by Tukey’s post hoc multiple pairwise comparisons (α = 0.05), and in **B** and **E** 2-sided *P* values were calculated by Student’s *t* test. *P < 0.05, **P < 0.01, ***P < 0.001, ***P < 0.0001.

We focused our follow-up in vivo therapy testing on the ATRi plus cisplatin combination, as it showed the strongest CI values across the tested ccRCC cell lines and other studies have identified this synergistic therapeutic combination in a range of other cancer cell lines from different tumor types (45–47). In other preclinical tumor models involving ATRi in combination with other DNA-damaging agents, best results are obtained when the DNA-damaging stimulus is given 18 hours prior to addition of an ATR inhibitor (48–50). We therefore conducted xenograft therapy studies using 786-O (Figure 7, A–C) and A498 (Figure 7, D–F) ccRCC cells growing as xenografts in Rag2 mice. Animals were treated once weekly for 3 (A498) or 4 (786-O) weeks with a single administration of cisplatin (2.0 or 2.5 mg/kg), a single administration of M4344 (10 mg/kg), or a single administration of cisplatin (2.0 or 2.5 mg/kg) followed 18 hours later by a single administration of M4344 (10 mg/kg). At the end of the treatment period in 786-O tumor–bearing mice, M4344 caused a 15% reduction, cisplatin a 43% reduction, and the combination therapy a 95% reduction in tumor volume compared with vehicle control–treated animals, indicative of in vivo synergy of this drug treatment. In A498 tumor–bearing mice, cisplatin and M4344 monotherapies caused tumor volume reductions of 4% and 20%, respectively, while combination therapy caused a 61% reduction in tumor volumes compared with controls, also indicating therapeutic synergy in this model. As expected from a potent chemotherapeutic agent that has known nephrotoxic effects in mice, the cisplatin- and M4344 + cisplatin–treated groups of mice lost weight during the therapy (Figure 7, C and F). In the 786-O cohorts, the weight loss was reversible upon cessation of therapy. These studies show that a weekly administration of cisplatin treatment followed by administration of ATRi effectively reduces tumor growth in 2 xenograft ccRCC models.

**Figure 8:**
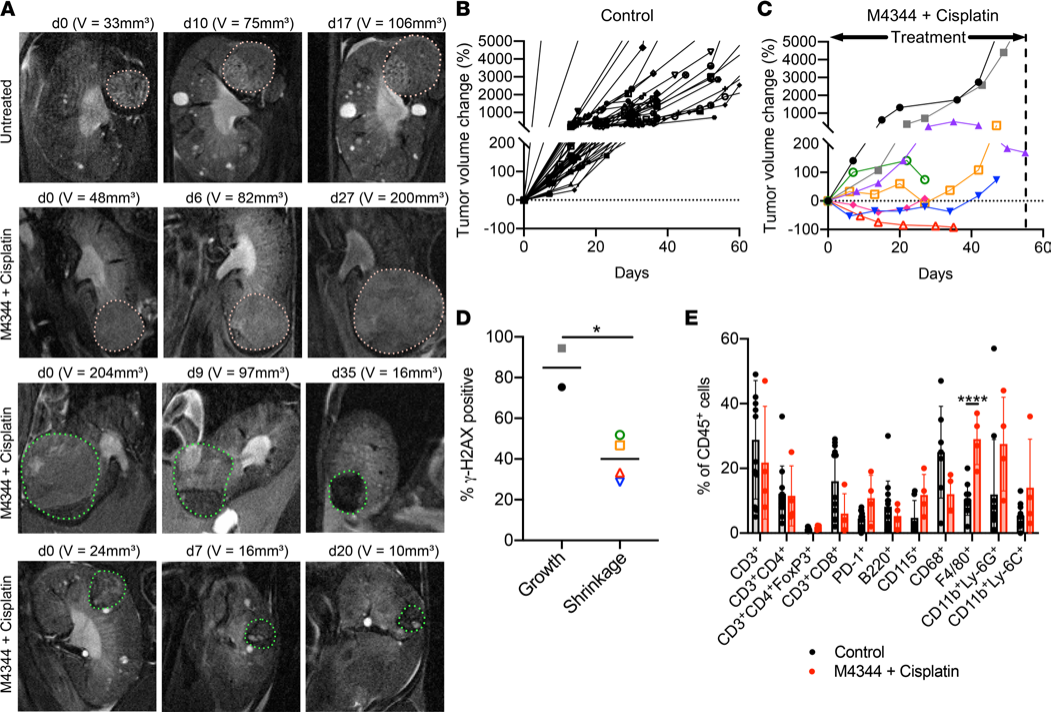
In vivo therapeutic efficacy of M4344 plus cisplatin in the *Vhl/Trp53/Rb1* mutant autochthonous ccRCC mouse model. (**A**) Representative MRI time course of ccRCC tumors (encircled) in an untreated mouse and 3 mice treated with weekly doses of cisplatin (2.5 mg/kg) followed by M4344 (10 mg/kg) 18 hours later. Days after therapy induction and tumor volumes are shown. (**B** and **C**) Relative *Vhl/Trp53/Rb1* mutant ccRCC tumor volume change over time in control mice (**B**) and M4344 + cisplatin–treated mice (**C**). Each line represents an individual tumor; volume changes were cut off at 5,000% for representation purposes. (**D**) Quantification of percentage of positively stained nuclei for γ-H2AX in tumors from **C** that showed continuous growth or that showed volume shrinkage as best response. Tumors were harvested 2 days after the final M4344 treatment. Tumors from **C** are identified in **D** by the corresponding symbol. Two-sided *P* value was calculated by Student’s paired *t* test, *P < 0.05. (**E**) Flow cytometric quantification of the cellular composition of the tumor immune microenvironment in control (*n* = 7–13) and M4344 + cisplatin–treated (*n* = 4) mice. Tumors were harvested 2 days after the final M4344 treatment. Data depict mean ± std. dev. *P* values were calculated using the 2-stage linear step-up procedure of Benjamini, Krieger, and Yekutieli, *q* = 1%, without assuming a consistent std. dev. between groups. ****P < 0.0001.

We next used the *Vhl/Trp53/Rb1* mutant autochthonous mouse ccRCC model to test this therapeutic regime in a more physiological context in the presence of an intact immune system. We have previously demonstrated that ccRCC tumors in this model show intertumor heterogeneity in therapeutic sensitivity to the antiangiogenic agent sunitinib and to the mTOR inhibitor everolimus (34), validating that the model reflects the variable interpatient sensitivities of human ccRCC to 2 clinically used therapies. We first demonstrated that 4 different mouse ccRCC cell lines showed patterns of sensitivity to ATMi, ATRi, DNA-PKi, and chemotherapeutic agents that were similar to the patterns observed in human ccRCC cell lines (Supplemental Figure 10A). Sublethal concentrations of M4344 (Supplemental Figure 10B) induced 3- to 4-fold reductions in the IC_50_ values of cisplatin and carboplatin treatment (Supplemental Figure 10C), indicative of therapeutic synergy, albeit of lesser magnitude than the synergy seen in human ccRCC cell lines. These findings provided the rationale for in vivo experiments: *Ksp-CreER^T2^ Vhl^fl/fl^ Trp53^fl/fl^ Rb1^fl/fl^* mice were fed tamoxifen for 2 weeks at 6 weeks of age and were monitored by ultrasound monthly beginning 5 months after tamoxifen feeding to identify tumor formation onset in each individual mouse (typically between 6 and 16 months after tamoxifen feeding). To provide accurate tumor volume determinations, mice were subsequently monitored on an approximately once- or twice-weekly basis by MRI once small tumors were detected (Figure 8A). Due to the labor- and time-intensive nature of these experiments, as well as the stochastic nature of the timing of tumor onset, which complicates the accrual of large cohorts of tumor-bearing mice, we did not conduct vehicle or single-treatment controls but rather compared the effect on tumor growth of a weekly administration of cisplatin (2.5 mg/kg) followed 18 hours later by a single administration of M4344 (10 mg/kg) to previously analyzed untreated mice (34, 51) as well as contemporaneously analyzed mice that were either untreated or that treated with IgG control antibodies in the context of another study that will be presented elsewhere. These mice represent a large pool of control mice with quantified tumor growth curves. We have previously shown that untreated tumors in this model follow exponential trajectories of tumor growth (51), and our control cohort also showed this relationship (Supplemental Figure 10D), while the M4344 plus cisplatin cohort did not (Supplemental Figure 10E). In the control group, 49 of 49 untreated tumors showed constant growth at every imaging (example in Figure 8A and quantification in Figure 8B). In contrast, in 4 mice treated with M4344 plus cisplatin, 2 tumors continued to grow under therapy while 3 showed reduction in tumor volume and 3 showed shrinkage after an initial period of growth (examples in Figure 8A and quantifications in Figure 8C). This study demonstrates that ATRi plus cisplatin therapy is effective in some, but not all, tumors in this autochthonous model of ccRCC. We next investigated whether the response of individual tumors correlated with the extent of DNA damage by staining for γ-H2AX. This analysis demonstrated that tumors that continued to grow during therapy had higher frequencies of γ-H2AX–positive cells than those tumors that showed a shrinkage of volume as best response during the period of therapy (Figure 8D).

Since the *Vhl/Trp53/Rb1* mutant autochthonous mouse ccRCC model is characterized by high infiltration of myeloid lineage cells and relatively low numbers of T cells (51) and given recent reports that ATRi and other DNA-damaging therapies can favorably modulate the tumor immune microenvironment toward an antitumor immunological state in other cancer models (52–54), we used flow cytometry to determine the relative fractions of different immune cells in control and M4344 plus cisplatin–treated tumors harvested 2 days after the final M4344 administration. Treatment did not alter the relative abundance of CD3^+^ T cells, CD4^+^ helper T cells, CD4^+^Foxp3^+^ regulatory T cells, CD8^+^ effector T cells, cells expressing the T cell activation and exhaustion marker PD-1, B220^+^ B cells, CD115^+^ blood monocytes, CD68^+^ monocytes/macrophages, CD11b^+^Ly6G^+^ neutrophils, or CD11b^+^Ly6C^+^ monocytes but increased the proportion of F4/80^+^ differentiated macrophages, potentially in response to the presence of dying tumor cells (Figure 8E). We conclude that in this ccRCC model ATRi plus cisplatin treatment does not induce an apparent immunogenic chemotherapy effect that improves T cell–mediated antitumor immunity.

## Discussion

TKIs and immune checkpoint therapies, as well as combinations of these agents, have transformed the therapeutic landscape for metastatic ccRCC and have greatly improved outcomes for many, but not all, patients (1, 55). Patients frequently discontinue these therapies due to intrinsic or acquired therapy resistance or for reasons of toxicity. New second-line, third-line, or later line therapeutic options, or alternative front-line therapies that offer better response rates and a higher percentage of durable regressions, are still needed. Here we identified inhibitors of the DNA damage–sensing ATR kinase as a potentially novel class of therapeutic drugs for ccRCC.

Previous studies have implicated pVHL as being necessary for efficient DNA damage signaling and repair (11–15). While not the primary focus of our study, our pVHL rescue studies do not suggest that restoration of pVHL to established *VHL*-null ccRCC cell lines affects responses to DNA-damaging agents. We used retroviral infection to reintroduce pVHL into 3 ccRCC cell lines, allowing the selection of populations of infected cells to avoid potential problems of clonal selection, and functionally validated suppression of HIF-α activity. We measured short-term (6 hours) and medium-term (24 hours) responses to DNA damage induction by doxorubicin by quantifying DNA damage foci (53BP1) and downstream DNA damage signaling (p-p53, p-ATM, p21) and observed no effects of pVHL rescue. Similarly, longer term (1 day to a week) responses (micronuclei formation, survival) to doxorubicin were also unaffected. Our 72-hour toxicity studies testing 7 chemotherapeutic agents, as well as molecular inhibitors of 3 DNA repair signaling molecules, also revealed no effect of pVHL reintroduction on the sensitivity of 3 ccRCC cell lines. We conclude that there are no strong and consistent effects of pVHL reintroduction on a variety of readouts of DNA damage. Our assays, however, did not directly address previously reported effects of pVHL reintroduction on cell cycle arrest and apoptosis induced by etoposide or adriamycin (11, 12), on DNA repair following γ-irradiation (13), or on clonogenic survival following γ-irradiation or PARP inhibition (14). It remains possible that pVHL regulates some aspects of the responses to specific forms of DNA damage in some cell lines. These reintroduction experiments into established cancer cell lines, which harbor multiple mutations, chromosomal alterations, and epigenetic changes, do not necessarily reflect the loss of function of pVHL in normal cells in the kidney at the beginning of tumor formation, meaning that at early stages of tumor development, pVHL loss may indeed lead to increased DNA damage (16) but that this defect becomes less pronounced as tumor evolution progresses and other mutations contribute to ongoing DNA damage or defects in DNA repair, irrespective of pVHL function.

In line with this argument, we found that human ccRCC cell lines and tumors, as well as cell lines and tumors from an autochthonous mouse model of ccRCC, exhibited high levels of endogenous DNA damage. This intrinsic DNA damage sensitized cells to ATRi. We developed potentially new assays that provide single-cell resolution of DNA damage responses across populations of cells, which permitted analyses of time- and concentration-dependent effects of ATRi. These analyses revealed that inhibition of the replication stress response was the primary mode of ATRi-induced DNA damage in 2 human ccRCC cell lines, accompanied by a dose-dependent increase in persistent DNA damage that was carried through into the G_2_/M and G_0_/G_1_ phases, leading to mitotic defects and the formation of micronuclei. All of these different consequences likely contribute to the antiproliferative and cytotoxic effects of ATRi. These findings are consistent with 2 recent studies that identified mRNA signatures of replication stress as a predictive factor for sensitivity of cancer cells to ATRi (39, 40). Our xenograft studies demonstrated that ATRi monotherapy inhibited ccRCC tumor growth in vivo. We further identified therapeutic synergies of ATRi with cisplatin, carboplatin, olaparib, and decitabine in human ccRCC cell lines, consistent with many preclinical studies in multiple tumor types involving multiple ATRi that identified synergy with cisplatin, carboplatin, and olaparib, as well as with γ-irradiation (30). Our findings open a new area of preclinical research in ccRCC that should aim to investigate and prioritize the most promising ATRi-based combination therapies for future clinical testing. In this context, we chose cisplatin as a proof of concept, as combination therapies with this agent are currently being tested in the clinic. We showed that weekly delivery of a single administration of cisplatin followed 18 hours later by a single administration of ATRi induced synergistic inhibition of tumor growth in 2 human ccRCC cell line xenograft models. Notably, the 786-O and A498 models were largely resistant to cisplatin monotherapy, analogously to the established resistance of ccRCC tumors to DNA-damaging chemotherapy, but the addition of ATRi sensitized these xenograft tumors to cisplatin. We believe that this provides strong preclinical evidence to suggest that ATRi might have a similar ability to sensitize human ccRCC to chemotherapy in the clinical context. Encouragingly, the combination of ATRi and cisplatin also showed therapeutic efficacy in most, but not all, tumors in the autochthonous *Vhl/Trp53/Rb1*-deficient mouse ccRCC model.

A very large body of preclinical and clinical research using a number of specific ATRi (including berzosertib, ceralasertib, and elimusertib) (comprehensively reviewed in ref. 30) has recently paved the way for potential clinical trials in ccRCC. At least 49 phase I and II clinical studies involving ATRi alone and in combination with many agents have been completed or are ongoing, and many of these have shown encouraging preliminary results (30, 48, 50, 56–59). These studies have already established that ATR inhibitor monotherapies are well tolerated and show clinical effects in some tumors (30). Given the antitumor effects of monotherapy with ATRi seen in our xenograft studies, we believe that clinical studies investigating ATR inhibitor monotherapy for immune checkpoint– and TKI-resistant ccRCC cases are warranted. Based on a large body of preclinical data, it is anticipated that combination therapies involving ATRi will likely show better antitumor effects than ATRi monotherapy. Berzosertib in particular has been successfully combined with DNA-damaging chemotherapy and has shown clinical benefit in 2 phase II studies, with some evidence of increased benefit in tumors with high replication stress (40). Specifically, the combination of berzosertib and gemcitabine improved progression-free survival in platinum-resistant high-grade serous ovarian carcinoma compared with gemcitabine alone in a randomized phase II study (60), and the combination of berzosertib and topotecan induced objective responses and durable tumor regressions in patients with relapsed small cell neuroendocrine cancers in a single-arm phase I/II study (40). As a potentially direct translation of our ATRi plus cisplatin combination therapy results, tolerated doses for berzosertib plus cisplatin (57) and berzosertib plus carboplatin (50) have already been established in phase I clinical studies. M4344, which was used in the current study, is no longer undergoing clinical development. However, the closely related, orally available compound M1774 has entered clinical development and is currently undergoing evaluation in a phase I study (ClinicalTrials.gov NCT04170153; ref. 61). It should also be noted that dose-limiting toxicities have been observed in some studies involving combination therapies with ATRi. Although the full picture of the extent of toxicities will emerge as more ongoing clinical trials report their results, it seems likely that ongoing and future clinical studies will need to continue to optimize dosing and scheduling to attempt to avoid or minimize toxicities while maintaining antitumor responses.

Until now, ccRCC has been excluded from all of these clinical studies, presumably largely due to the established clinical resistance of this tumor type to chemotherapy. It is hoped that our current study will alter the prevailing view that DNA damage and DNA damaging signaling networks are not attractive therapeutic targets for ccRCC. It is possible that other combination strategies will be able to be identified that take advantage of molecular inhibition of other key players in DNA sensing and repair pathways, potentially in combination with specific ccRCC tumor genotypes. It would moreover be desirable to identify therapeutic combinations that do not rely on indiscriminate induction of DNA damage by conventional chemotherapeutic agents, which causes systemic toxicities, but rather that induce DNA damage more specifically in the context of altered genetic or phenotypic features of ccRCC cancer cells. An excellent example of this concept was the discovery that inhibition of glutaminase 1 (GLS1) in ccRCC cells leads to metabolic alterations that induce reactive oxygen species–mediated DNA damage (62). This leads to a therapeutic synergy between GLS1 inhibition and inhibition of PARP enzymes with olaparib (62).

Our experiments also suggest that there is likely to be some degree of heterogeneity between different tumors in terms of responses to a possible future ATRi plus cisplatin therapy. While this drug combination exhibited synergy in all 5 human ccRCC cell lines tested, we observed some variability in the CI values, indicative of the strength of synergy, between the cell lines. We also observed heterogeneity in the therapeutic sensitivity of ccRCC tumors in the autochthonous model, consistent with our previous observations of therapeutic heterogeneity with sunitinib and everolimus (34), and with our previous exome sequencing, which showed that individual tumors in this model displayed different sets of genetic mutations. For example, 2 out of 7 tumors exhibited amplification of the *Myc* gene (34), a known ccRCC driver. Thus, genetic inter- and intratumor heterogeneity in ccRCC may sensitize or induce relative resistance to ATRi or ATRi plus cisplatin therapies. Our mouse model represents one experimental system that could be used to address this issue in future larger scale studies coupling therapy to genomic analyses of responder and nonresponder tumors. Our preliminary data from the mouse model surprisingly suggest that higher levels of DNA damage correlate with resistance to ATRi plus cisplatin therapy. While this is seemingly counterintuitive as it might be expected that inhibiting DNA damage sensing through ATR inhibition would have stronger effects in cells with high levels of DNA damage, we speculate that tumors with high levels of DNA damage may have evolved to tolerate this DNA damage and are therefore more resistant to induction of further damage than those tumors with lower levels of endogenous DNA damage. Experimental genetic manipulation of other commonly mutated ccRCC genes, such as *PBRM1*, *BAP1*, *SETD2*, *KDM5C*, *TP53*, and other DNA repair genes, in cell culture models could also be conducted to investigate potential ATRi therapy–based modifying effects of these genes or to determine whether specific mutations may lead to sensitivities to other forms of pharmacological manipulation of DNA repair networks. It is hoped that these types of studies will pave the way for future genotype-dependent, personalized therapies based on a new generation of molecularly targeted DNA damage–inducing therapies in ccRCC.

## Methods

A full description of the methods used in this study is provided in Supplemental Methods.

### Data sets generated in this study.

Fastq files of RNA sequencing and whole-exome sequencing have been uploaded to SRA and are accessible through BioProject PRJNA870454 (https://www.ncbi.nlm.nih.gov/sra/PRJNA870454). Raw counts of RNA-sequencing data can be found at the Gene Expression Omnibus with the identifier GSE211466 (https://www.ncbi.nlm.nih.gov/geo/query/acc.cgi?acc=GSE211466).

### Study approval.

Human ccRCC samples from partial or complete nephrectomies were collected at the Institute for Surgical Pathology, Medical Center – University of Freiburg. All patients provided written informed consent, and the study was carried out under Ethik-Votum 403/20 of the Medical Center – University of Freiburg. Autochthonous mouse tumor model studies were conducted under permission G-17/112 of the Regierungspräsidium Freiburg, Germany. Mouse xenograft studies were conducted under protocol registration numbers DA4/Anz.1014 and DA4/Anz.1040 of the Regierungspräsidium Darmstadt, Germany.

## Author contributions

PS, RKL, FTZ, JA, and IJF conceptualized the study; PS, DVE, WR, MF, and IJF developed methodology; PS, KZ, PM, and MB performed formal analysis; PS, AR, KZ, AP, FN, YF, HH, RH, MA, CW, and DJH investigated; CS provided resources; IJF wrote the original draft; PS, AR, AP, KZ, PM, RH, RKL, FTZ, DVE, WR, CS, MF, and JA reviewed and edited the draft; PS, AR, AP, and IJF visualized data; JA and IJF supervised the study; and IJF acquired funding. Co–first authorship was shared by PS, AR, KZ, and AP, with the order determined by the overall contribution to final figures and time investment.

## Supplementary Material

Supplemental data

Supplemental data set 1

Supplemental data set 2

Supplemental data set 3

Supplemental data set 4

Supplemental data set 5

## Version 1 11/22/2022

In-Press Preview

## Version 2 12/22/2022

Electronic publication
