# Supplementary material for: ATR represents a therapeutic vulnerability in clear cell renal cell carcinoma
Source: JCI Insight. 2022 Dec 22;7(24):e156087. doi: 10.1172/jci.insight.156087 (PMC9869969; doi:10.1172/jci.insight.156087)

Supplementary Methods

Cell culture

Cell lines used in this study were 786-O, 769-P, A498 (all from Wilhelm Krek, ETH Zurich), RCC4 (ECACC), SLR22 (from Holger Moch, University Hospital Zurich), RKO (from Nils Blüthgen, Berlin), A549, HCT116, H1299, HEK293 (all from ATCC) as well as human primary RPTEC (ATCC). Tumour cell lines were cultured in DMEM (Gibco) + 10% FCS (Sigma) + 1% Penicillin / Streptomycin (Gibco). VHL-expressing cells and their respective vector controls were derived by infecting target cells with empty or VHL30-expressing retrovirus (pCMV(R)-Neo). These cells were additionally cultured with Geneticin (Gibco) to ensure stability of the expression vector. Mouse ccRCC cell lines were isolated from a piece of tumour tissue from tamoxifen-treated *Ksp-CreER*^{T2}*; Vhl*$^{fl/fl}$*; Trp53*$^{fl/fl}$*; Rb1*$^{fl/fl}$ mice (1), minced with a scalpel blade and digested for 70 minutes at 37°C with 1 mg/ml Collagenase II solution in 1x HBSS. The digestion was inactivated with 20 ml K1 medium (Dulbecco's modified Eagle's medium (DMEM) and Hams F12 mixed 1:1, 2 mM glutamine, 10 kU/ml penicillin, 10 mg/ml streptomycin, hormone mix (5 µg/ml insulin, 1.25 ng/ml prostaglandin E_1 (PGE_1), 34 pg/ml triiodothyronine (T3), 5µg/ml transferrin, 1.73 ng/ml sodium selenite, 18 ng/ml of hydrocortisone and 25 ng/ml epidermal growth factor (EGF)) + 10% FCS). The cell solution was subsequentially filtered through a 70-µm cell strainer, pelleted, and cultured in K-1 medium + 0.5% FCS in a humidified 5% (v/v) CO_2 and 20% O_2 incubator at 37°C. Primary mouse renal epithelial cells were isolated from non-Cre kidneys of tamoxifen-treated *Vhl*$^{fl/fl}$*; Trp53*$^{fl/fl}$*; Rb1*$^{fl/fl}$ mice by dissecting the kidneys and removing the capsula and surrounding adipose tissue. The kidneys were minced with a scalpel blade in 10 ml of collagenase II (1 mg/ml) and soybean trypsin inhibitor (1 mg/ml) in HBSS and the digestion mixture transferred to a bottle which was put in a beaker with water at 37 °C and incubated for 30 min. The mixture was filtered through a 70-µm cell strainer 20 ml of wash solution (5 % FCS in HBSS) was added. The cells were spun down at 500 x g for 5 min and the supernatant aspirated followed by two further washes in wash solution and one wash in K1 + 0.5% FCS. The cell pellet was subsequently resuspended in 1 ml ACK lysing buffer for around 1 min to deplete the red blood cells. After 1 minute, ACK lysing buffer was diluted in 10 ml K1 medium + 0.5% FCS and the cells centrifuged. Finally the cells were resuspended in 10 ml K1 medium + 0.5% FCS and cultured in a cell culture dish until full confluency.

Drugs

The following drugs were dissolved in DMSO and stored as aliquots at -80°C: Berzosertib (M6620), M4344, M3541, M4076, peposertib (M3814) (all from the healthcare business of Merck KGaA, Darmstadt, Germany), carboplatin (MedChemExpress, HY-17393), cisplatin (MedChemExpress, HY-17394), oxaliplatin (MedChemExpress, HY-17371), decitabine (MedChemExpress, HY-17371), fluorouracil (MedChemExpress, HY-90006), acriflavine (Sigma, A8126), doxorubicin (MedChemExpress, HY-15142), paclitaxel (MedChemExpress, HY-B0015), vincristine (MedChemExpress, HY-N0488A), olaparib (MedChemExpress, HY10162), camptothecin (MedChemExpress, HY-16560), irinotecan (MedChemExpress, HY-16562), etoposide (MedChemExpress, HY-13629), hydroxyurea (MedChemExpress, B0313).

Survival and drug synergy assays

For survival assays, drug dilutions were first prepared in 96-well plates in normal cell culture medium in a 2-fold concentration compared to the final concentration (volume of 100 µL). 5000 cells per well were then plated

in 100 µL, therefore diluting drug to the desired final concentration. Cells were grown for 72 hours and fixed and stained using a Crystal Violet (0.1%) solution in 80% ddH20 and 20% EtOH for 10 minutes at room temperature. Plates were then extensively washed and dried. For quantification, a solution consisting of 50% EtOH and 0.5% Triton X100 was added in order to dissolve the crystal violet. Plates were then measured in a Tecan Spark Multimode Microplate Reader at 590 nm. In order to handle the high number of microplates (100 – 200), we evaluated the results using Python Data Science tools including scipy and matplotlib, and the scripting is documented in Jupyter Notebooks (see attachments). Raw data was normalised using background intensities of wells without cells and wells without drug treatment (range of 0 – 1). To determine the curve fit, fitting statistics and IC50, we employed a sigmoidal mathematical model and the curve-fitting algorithm of scipy. For single dose synergy treatments, we added sublethal concentrations of the Merck drugs with complete dilution series of the other genotoxic substances. We determined the IC50 for the combination and normalised it to the IC50 of the substance's single treatment. For full synergy assays, 2,000 cells per well were seeded in 96 well plates, 24 hours after seeding, medium was changed and after another 24 hours (day 0) cells were treated with different concentrations of the inhibitors for three days. Concentration range was chosen for each drug according to previous empirically-determined IC50 values and applying a dilution factor of 2. After completion of the drug exposure, cells were analysed using the sulforhodamine B (SRB) colorimetric assay as described (2). Absorbance values were used to determine the coefficient of drug interaction (CI) with the Anaconda® 5.3_jupyter 5.6.0 software. The software calculates and plots drug-response and drug interaction curves by using the method of isoboles. Dose-response curves (ellipsoid) were obtained according to the formula b = B*(1 - (a/A)**n)**(1/n) where a and b are the doses of Drug A and Drug B respectively when the two are present together, A and B are the respective individual doses for that effect level (IC50) and n is curvature (n = 1 additive, n < 1 super-additive (also known as synergistic), n > 1 sub-additive) (3).

Immunohistochemistry

Immunohistochemical staining of formalin-fixed paraffin embedded tissues was conducted as described (4) using anti-phospho-Ser139 histone H2A.X (γ-H2AX) (Cell Signaling Technology, #9718, 1:300) and antigen retrieval with citrate buffer for 15 min at 110ºC. Percentage of positive nuclei was quantified using QuPath software.

Immunofluorescence microscopy – Conventional staining

Cells were cultured on glass coverslips (circumference 13 mm; in 24-well plates). For fixation, these cells were washed with PBS and incubated with 4% PFA (Sigma) for 10 minutes at room temperature. After washing with PBS to remove residual PFA, cells were blocked and permeabilised with PBS + 3% BSA (Sigma) + 0.1% Triton-X100 for 30 minutes at 4°C. PBS + 0.6% BSA + 0.02% Triton-X100 was used for primary and secondary antibody incubations. Primary antibodies were incubated overnight at 4°C, while secondary antibodies were incubated for 2 hours at room temperature. After antibody incubations, cells were incubated with PBS + DAPI (Thermo Fisher, 1µg/ml) and washed three times with PBS. After completion of the staining, coverslips were fixed on microscopy object slides using Fluoromount-G (Thermo Fisher). The following antibodies were employed: anti-53BP1 (Abcam, Ab36823, 1:500), anti-p21 (Cell Signaling Technology, CST2947, 1:200), anti-phospho-S1981-ATM (Cell Signaling Technology, CST4526, 1:200), anti-phospho-S15-p53 (Cell Signaling Technology, CST9284, 1:100), goat anti-rabbit Alexa 568 (Thermo Fisher, A11011, 1:600), goat anti-mouse

Alexa 568 (Thermo Fisher, A-11031, 1:600), goat anti-rabbit Alexa 647 (Thermo Fisher, A-21244, 1:600), goat anti-mouse Alexa 647 (Thermo Fisher, A-21235, 1:600).

Immunofluorescence microscopy – Multiplex staining

Procedures in multiplex microscopy were adapted to allow image acquisition on immersed samples (5). Cells were grown in 96-well imaging plates (Corning; # 353219). The plates were washed in PBS and fixed with 4% PFA for 10 minutes at room temperature. The staining cycle consisted of the steps of sample blocking and antibody staining as for conventional microscopy, plus the additional steps of image acquisition and antibody elution. During sample blocking, PBS + 3% BSA + 0.1% Triton-X100 was supplemented with 150mM Maleimide which binds and blocks free sulfhydryl groups. The antibody staining was as for the standard protocol above. To prevent photo-crosslinking, image acquisition was performed with samples immersed in PBS + 700mM N-Acetyl-Cysteine as a radical scavenger. Antibody elution was done for 10 minutes at room temperature with an elution buffer consisting of 0.5M L-Glycine, 3M Urea, 3M Guanidinum chloride (GC) and 70mM TCEP-HCl (TCEP) in ddH_20 set to pH 2.5.

EdU cell-cycle analysis

For EdU incorporation and staining, we made use of the Click-It EdU Alexa Fluor 647 assay kit (Thermo Fisher, C10424). For EdU incorporation, 20 µM EdU was prepared in fresh cell culture medium, pre-warmed to 37°C and added to the cell culture for 30 minutes at 37°C. Cells were then re-suspended in normal cell culture medium for recovery. Fixation of cells was performed depending on the downstream application (4% PFA for microscopy; 70% EtOH for flow cytometry). Fixed cells were blocked and permeabilised with PBS + 3% BSA + 0.1% TX100 for 30 minutes at 4°C. EdU staining solution including copper sulfate and azide-coupled Alexa Fluor 647 was prepared according to the manufacturer's instructions and added to the cells for 30 minutes at room temperature. Cells were then washed with PBS. When combined with antibody stains, the antibody incubation was performed after the EdU staining. For multiplex microscopy, EdU staining was performed during the final staining cycle.

Replication stress test

For assaying progression through S-phase, cells were first labelled with EdU for 30 minutes as described above. EdU was removed and one batch of cells were trypsinised, fixed in 70% ice-cold EtOH and stored at -20°C. Another batch was left in complete medium for recovery for 6 hours and then also fixed. We then performed an EdU staining as described above and counterstained with 5µg/ml DAPI for 10 minutes. Flow cytometry was done on a BD LSR Fortessa flow cytometer. Living single cells were then gated into four distinct populations according to DAPI and EdU: G0/G1 cells (DAPI 2n; EdU low), S-phase cells (DAPI 2n – 4n; EdU high), G2-phase cells (DAPI 4n; EdU low) and post-mitotic EdU-labelled cells (DAPI 2n; EdU intermediate). From all these subpopulations, the cell numbers and mean DAPI intensity were recorded. For analysis, the aim was determining how far EdU-labelled cells had progressed in DNA-replication since the EdU pulse. For this, we determined the DNA content at 6 hours and at 0 hours after EdU pulse. Post-mitotic EdU-labelled cells have completed DNA-replication and therefore are assigned a genome set of 4n. Because two post-mitotic cells are derived from one cell that was originally labelled, the contribution of that subpopulation is weighted with half its cell number. For the subpopulation of S-phase cells, their mean DAPI value is normalised to G0/G1 and G2 subpopulations (value between 2n and 4n) and contributes with the number of cells of this

subpopulation. Zero hour (post-EdU) values were then subtracted from 6 hour values to account for different DNA content induced by pre-treatment.

Average DNA content of S-phase population = [(0.5 x Cell numbers (post mitotic) x 4n) + (Cell numbers (S) x n(S))] / [Cell numbers (post mitotic) + Cell numbers (S)]

Replication Progress (sample) = DNA content (6 hours) – DNA content (0 hours)

DNA fibre assay

SLR22 and 786-O cells were seeded 24 h prior to experiment and then treated for 24 h with 200 nM M4344. The cells were pulse-labelled as indicated with 5 mM 5-iodo-2'-deoxyuridine (IdU, Fisher Scientific 11473040) followed by 500 mM of 5-chloro-2'-deoxyuridine (CldU, Sigma Aldrich C6891) for 20 min each. Cells were collected and resuspended in PBS at a density of 1 x 10^6 cells/ml. 2 µl of cell suspension was spotted onto a Superfrost microscope slide (Thermo Fisher) and dried for 7 min. The spots were overlaid with 7 µl of DNA lysis buffer (200 mM Tris-HCl, pH 7.5, 50 mM EDTA, 0.5% SDS). After 5 min, the slides were tilted by 15 degrees to allow the lysates to slowly move down the slide. The DNA fibres were air-dried for 4 h, fixed in a 3:1 solution of methanol/acetic acid for 10 min at RT, air-dried and stored at 4°C overnight. The slides were then washed twice in distilled water and denatured with 2.5 M HCl for 60 min at RT, washed three times in PBS and incubated for 20 min in blocking buffer (5%BSA/PBS) at RT. The slides were incubated with a mouse anti-BrdU antibody (to detect IdU) (Becton Dickson, 1:40, 347580) and a rat anti-BrdU antibody (to detect CldU) (Abcam, 1:40, ab6326) for 2 hr at RT. Slides were then washed three times in PBS followed by incubation with Alexa Fluor 488-conjugated anti-rat (1:200) and Alexa Fluor 568-conjugated anti-mouse (1:200) for 1h at RT. The slides were then washed three times in PBS and mounted in Vectashield anti-fade mounting media (Biozol). Fibre images were obtained with a Zeiss LSM880 microscope using a 63X objective. Pictures were processed with AutoQuantX3.1 deconvolution software and analysed with ImageJ. In each assay 50-100 fibres were measured and experiments were repeated three times.

Microscopy Imaging

All image acquisitions were done on an Olympus scanR IX-81-based HCS microscope system using the UPLSAPO 20X objective. To allow quantitative analysis, regions of 3x3 – 5x5 fields of view (300 – 1000 single cells) were recorded. To allow accurate detection of pan-nuclear signals and DNA-damage foci in different imaging planes, acquisition was done with z-stacks with a 5µm range around the autofocus plane.

Microscopy Image Processing and Analysis

Image processing was performed using functions and plugins from ImageJ. To allow automatic batch processing, scripts were generated in ImageJ macro language (attachments). For image processing, raw images consisting of z-stack image planes were background subtracted and aggregated by maximum projection. Image tiles were recombined by Grid/Collection stitching. To combine the different channels in multiplex imaging experiments, we used Descriptor-based registration (2d/3d). For segmentation of nuclei and micronuclei, the registered images were first corrected by subtraction of a strongly Gaussian blurred (sigma=30) image copy. This procedure allows sufficient edge detection for the small micronuclei in close vicinity of nuclei. Following standard automatic thresholding and mask conversion, micronuclei and nuclei were detected with particle analysis. Nuclear intensities in different image channels (DAPI, EdU, pATM) were then extracted with single-cell resolution. For focus analysis, 53BP1 and pATR channels were corrected with a

strong rolling-ball background subtraction (radius=10 pixels) to remove nuclear background and highlight focus areas. DNA-damage foci were then quantified via particle analysis. For data analysis we employed Python tools for data science including scipy, pandas and matplotlib. All analysis was documented in Jupyter notebook scripts (see attachment). First, nuclei and micronuclei were classified by size and by DAPI integrated intensities employing the DBSCAN cluster algorithm. All channels were again corrected for residual illumination defects and mean normalised. Cell-cycle classification was done by DAPI and EdU integrated intensities. Mitotic cells were identified by high chromatin densities using DAPI modal values. For all pan-nuclear quantifications, data was additionally normalised to non-treated controls. To find an appropriate norm for DNA-damage focus quantification, which takes into account the considerable size differences of single foci, we took the logarithm of single focus intensities, summed them up and normalised to cell numbers. Alternatively, we showed focus formation including a second dimension for the variation in focus intensities. For micronucleus analysis, we assigned micronuclei to neighbouring nuclei with a maximum distance of 60 pixels, and then quantified the fraction of micronucleus-containing cells within the population. We also performed all these quantifications in a cell-cycle dependent fashion.

Western Blotting

For sample preparation, treated cells were washed once with ice-cold PBS and lysed in RIPA buffer + Protease Inhibitors (NaVO3, PMSF, NaF, PI cocktail Roche) and DTT. Solutions were then sonicated with a Bandelin Sonopuls (on ice, 20 seconds at 100%) and insoluble material was removed by centrifugation. Protein concentrations were determined with a BCA assay (ThermoFisher). SDS-PAGE was performed under standard conditions. We employed a Tris-Glycine transfer buffer with 15% methanol and 0.05% SDS using nitrocellulose membrane (Bio-Rad) in a wet blotting system (Bio-Rad). Membranes were blocked with TBS-Tween (0.1%) with 5% non-fat dry milk (Sigma) for 1 hour at room temperature before each antibody incubation. Antibody solutions were prepared in TBST + 3% BSA (Sigma) and incubated overnight at 4°C (primary antibodies) or for 2 hours at room temperature (secondary antibodies). The following antibodies were used: anti-VHL (Cell Signaling Technology, CST68547, 1:1,000 dilution), anti-VINCULIN (Abcam, ab130007, 1:500), anti-VINCULIN (Santa Cruz, sc-25336, 1:1,000), anti-ß-actin (Sigma-Aldrich, A2228, 1:5,000), anti-NDRG1 (Cell Signaling Technology, CST5196, 1:1,000), anti-CyclinD1 (Cell Signaling Technology, CST55506, 1:1,000), anti-Glut1 (Abcam, ab14683, 1:500), anti-CaIX (Thermo Fisher, PA1-16592, 1:2,000), anti-Hif1a (Novius, NB100-479, 1:500), anti-Hif2a (GeneTex, GTX30114, 1:1,000), anti-ATR (Cell Signaling Technology, CST13934, 1:1,000), anti-phospho-ATR (Cell Signaling Technology, CST2853, 1:1,000), anti-ATM (Cell Signaling Technology, CST2873, 1:1,000), anti-phospho-ATM (R&D Systems, S1981, 1 µg/ml), anti-phospho-Chk1 (Cell Signaling Technology, CST2348, 1:1,000), anti-gH2AX (Cell Signaling Technology, CST9718, 1:1,000), anti-53BP1 (Abcam, ab36823, 1:10,000), anti-p21 (Cell Signaling Technology, CST2947, 1:1,000), anti-phospho-p53 (Cell Signaling Technology, CST2526, 1:1,000), anti-PARP (Cell Signaling Technology, CST9532, 1:1,000), anti-Caspase3 (Cell Signaling Technology, CST9662), goat-anti-rabbit IgG (H+L) HRP-conjugated (Thermo Scientific, 31460, 1:10,000), goat anti-rabbit IgG IRDye 800CW (LI-COR, 926-32211, 1:10,000), goat anti-rabbit IgG IRDye 680RD (LI-COR, 926-68071, 1:10,000). Membranes were washed three times for 10 minutes with TBST after the antibody incubations. The LI-COR Odyssey Imaging System or ECL were used to detect bound antibodies.

Whole exome sequencing analysis

DNA was isolated from cell lines and whole exome sequencing was performed on an Illumina HiSeq 4000 System (paired-end 100bp) by the core facility of the DKFZ in Heidelberg with the LI Exom v6+UTR exon library preparation kit. Raw data fastq-files were pre-processed with trimmomatic (version 0.38) (6) to assure sufficient read quality by removing adapters and low quality bases with a quality score below 10 at the end of the reads (ILLUMINACLIP:2:30:10 HEADCROP:3 TRAILING:10 MINLEN:25). The overall quality of the bases and reads was good, with an average of 99% of the raw reads surviving the trimming step. After quality control and trimming, the reads were aligned to the hg19 reference genome from UCSC using the BWA-MEM algorithm (version 0.7.17-r1188) (7). Somatic mutations were called following the GATK Best Practices workflow for somatic short variant discovery (SNVs + indels) (https://gatk.broadinstitute.org/hc/en-us/articles/360035894731-Somatic-short-variant-discovery-SNVs-Indels-) using the tool gatk4 (version 4.1.4.0). Variants were called using Mutect2 (https://gatk.broadinstitute.org/hc/en-us/articles/360036713111-Mutect2) with the tumour-only mode and filtered based on the Variant Allele Frequency (VAF) > 10%, Minor Allele Frequency (MAF) < 0.1%, and minimum coverage of 4 with at least 2 reads supporting the alternate allele. Only exonic variants were kept while removing synonymous mutations. The filtered variants were annotated with ANNOVAR (version 2020-06-08) (8) and SnpEff (version 4.3t) (9), Supporting Information File 3 contains the filtered variants. Oncoprints were generated using a modified version of the function TCGAvisualize_oncoprint from the R/Bioconductor package TCGAbiolinks (version 2.18.0) (10). Raw exome sequencing data have been uploaded to SRA and are accessible through BioProject PRJNA870454 (https://www.ncbi.nlm.nih.gov/sra/PRJNA870454).

RNA sequencing

RNA was isolated from cell lines using the NucleoSpin RNA kit (Macherey Nagel). Paired-end RNA-sequencing was performed on an Illumina NovaSeq 6000 System (flow cell type S1 for 2 x 100bp) for the human samples and on an Illumina HiSeq 4000 System (100bp) for the murine samples by the core facility of the DKFZ (German Cancer Research Center) in Heidelberg with the Illumina TruSeq Stranded RNA library preparation kit. Raw data fastq-files were pre-processed with trimmomatic (version 0.38) (6) to assure sufficient read quality by removing adapters and low quality bases with a quality score below 10 at the end of the reads (ILLUMINACLIP:2:30:10 HEADCROP:3 TRAILING:10 MINLEN:25). The overall quality of the bases and reads was good, with an average of 97% of the raw reads surviving the trimming step. After quality control and trimming, the human reads were aligned to the GRCh37 and the murine reads to the GRCm38 reference genome from Ensembl using the STAR aligner (version 2.5.2b) (11). 93.36% ± 0.41% of the human reads and 90.17% ± 0.57% of the murine reads were uniquely mapped and considered. Using the R/Bioconductor package DESeq2 (version 1.30.0) (12), the raw read counts were normalized (normCounts) and transformed to the $\log_2$ scale (rlogCounts), to compensate for a large dynamic range of expression values, by considering the library size. Additionally, lowly expressed genes were removed. For the human samples, only genes with normalised counts ≥ 10 in at least 75% of the samples were kept. For the murine samples, only genes with at least 5 counts across all 9 samples were kept. For the remaining 13,742 human genes, log2 fold changes (log2FC) between each ccRCC cell line and the RPTEC control were calculated using the formula:

$$log2FC = log2(\frac{normCounts\_ccRCC + 1}{normCounts\_RPTEC + 1})$$

The remaining 23,917 murine genes, were fitted with a negative binomial generalized linear model followed by Wald statistics using the R package DESeq2 to obtain log2FC between the pooled tumour samples versus

pooled normal samples. Supporting Information Files 1 and 2 contain log2FC, normCounts and rlogCounts for the different datasets. Raw RNA sequencing data have been uploaded to GEO with the identifier GSE211466 (https://www.ncbi.nlm.nih.gov/geo/query/acc.cgi?acc=GSE211466).

TCGA analysis

Publicly available raw RNA-seq data of the TCGA (The Cancer Genome Atlas) KIRC (Kidney renal clear cell carcinoma) samples were downloaded from the NCI (National Cancer Institute) GDC (Genomic Data Commons) (13) using the R/Bioconductor package TCGABiolinks (version 2.18.0) (10) (project: TCGA-*TumourType*, data category: Gene expression, data type: Gene expression quantification, experimental strategy: RNA-seq, platform: Illumina HiSeq; file type: results, legacy: TRUE; downloaded on 02.03.2021 (KIRC) and 24.11.2021 (other tumour types). In a first analysis, the read counts of the complete dataset, including 72 solid tissue normal samples and 533 primary tumour samples for KIRC, were normalised, lowly expressed genes were removed, and a variance stabilizing transformation was applied using the R/Bioconductor package DESeq2. In a second analysis, only the 72 solid normal tissue samples and their matching 72 primary tumour samples from the same patient were used to calculate log2FC between the pooled tumour samples versus pooled normal samples using DESeq2. For 336 tumour samples, mutation annotations were available in the GDC data portal and downloaded using the function GDCquery_Maf (tumour = KIRC, pipelines = mutect2; downloaded on 03.03.2021) from TCGABiolinks. Of these, only one sample did not have expression data and was therefore excluded.

Gene Set Enrichment Analysis (GSEA)

Enrichment of signalling pathways was performed on the rlogCounts from both the human and mouse ccRCC RNA-seq data as implemented in the R/Bioconductor package gage (Generally Applicable Gene-Set Analysis) (version 2.40.1) (14) with signalling pathways from Gene Ontology (15, 16) and MSigDB (17) (human gene sets downloaded on 16.11.2020, murine gene sets downloaded on 26.01.2021). Statistics were computed using the Kolmogorov-Smirnov test and pathways were considered significant with an adjusted P value < 0.05 (Benjamini-Hochberg). The overlap of significant pathways between the human and mouse datasets was determined while considering only pathways that were found in both species. Supporting Information File 4 contains the overlap of significantly up-regulated MSigDB Reactome pathways between the human and mouse ccRCC dataset. UpSetR intersection plots were generated using a modified version of the R package UpSetR (version 1.4.0) (18).

Enrichment plots

Based on the log2FC, all genes were ranked for each RNA-seq dataset, including the human ccRCC cell lines, the mouse ccRCC cell lines, ccRCC tumour samples from VpR mice (1), and the TCGA KIRC samples. Enrichment plots were generated for specific Reactome pathways associated with DNA damage repair using the GSEA_fgsea function from the R/Bioconductor package DOSE (Disease Ontology Semantic and Enrichment analysis) (version 3.16.0) (19) and the gseaplot2 function from the R/Bioconductor package enrichplot (version 1.10.2). P values were adjusted using the Benjamini-Hochberg correction.

Single-sample GSEA (ssGSEA)

ssGSEA (20) was used to assess the heterogeneity of specific DNA damage repair responses and a proliferation signature (21) in TCGA KIRC samples based on the log2 transformed expression values. For each pathway in each TCGA KIRC sample, the R implementation ssGSEA2.0 (22) was used to calculate normalised enrichment scores (NES) and *P* values (Supporting Information File 5) using permutations.

ccRCC xenograft assays

In vivo efficacy data were generated in human ccRCC xenograft models 786-O (ATCC, CRL-1932 ™) and A498 (ATCC, HTB-44™). Eight- to ten-week-old female H2d Rag2 [C;129P2-H2^{d}-TgH(Il2rg)^{tm1Brn}-TgH(Rag2)^{tm1Alt}N4] mice (Taconic Biosciences, Denmark) were used. 2 × 10^6 tumor cells (786-O) and 5 × 10^6 tumor cells (A498) were injected subcutaneously in the flank, in 100 µl Dulbecco's phosphate-buffered saline. When tumor xenografts reached a mean volume of 100 to 200 mm^3, mice (N = 6-10 per treatment arm, randomized from 15 mice per arm to obtain a similar mean and median within the treatment groups) received the respective treatment. M4344 was suspended for oral administration in a vehicle of 15% Captisol in water solution, pH 4.75 and administered via oral gavage at a volume of 10 mL/kg. Cisplatin was administered intraperitoneally at the indicated doses as a solution in 0.9% saline. Tumor length (L) and width (W) were measured with calipers and tumor volumes were calculated using $L \times W^2/2$. For statistical analysis of treatment effects on tumor growth, tumor volume data were log-transformed and analyzed by repeated measures analysis of variance (RM-ANOVA) followed by Tukey's post-hoc multiple pairwise comparisons (α=0.05) using GraphPad Prism 8 software. Student's t-test was used to investigate statistically significant differences of tumor volumes between 2 groups. Statistically significant differences are labeled with * $p<0.05$, ** $p<0.01$, *** $p<0.001$, **** $p<0.0001$.

Autochthonous ccRCC tumour studies

Gene deletion in 6 week-old mice Ksp1.3-CreER^{T2};*Vhl*$^{fl/fl}$;*Trp53*$^{fl/fl}$;*Rb1*$^{fl/fl}$ mice (1) was achieved by feeding with food containing 400 mg/kg tamoxifen citrate (Ssniff, diet A155T70401) for 2 weeks. Kidneys were monitored on a monthly basis beginning 5 months after feeding using ultrasound imaging. Hair from the area of the kidneys was removed from anaesthetised mice (2.5% Isofluorane by inhalation for induction, 1.5% for maintenance) using clippers followed by depilatory cream (Veet for Men). Ultrasound gel was applied and kidneys imaged using a VeVo3100 imager. MRI was conducted using a 9.4 tesla small bore animal scanner (BioSpec 94/21, Bruker Biospin, Ettlingen, Germany) and a mouse quadrature-resonator (Bruker, Ettlingen, Germany). Mice were anaesthetized and heart and respiration rate were constantly monitored to avoid artefacts. The MRI involved a localiser sequence as a pilot scan to localise the kidneys followed by a coronal oriented T2-weighted spin echo RARE (Rapid Acquisition with Relaxation Enhancement) sequence over the kidneys and bladder. This sequence was performed to delineate the tumor from the surrounding healthy tissue. The RARE sequence in coronal orientation featured a FOV of 30 mm x 30 mm; a matrix size of 256 x 256 $pixel^2$, and an in-plane resolution of 117 x 117 μm^2. The slice thickness was 0,50 mm with no slice spacing to achieve contiguous image sets of the whole volume (TR/TEeff/FA: 3800ms/40ms/180°). The number of slices was adjusted to the measured volume (on average 30). The circumference of each tumor was manually annotated on each MRI-section using MIPAV software (v8.0.2). Individual tumor volumes were then calculated using the inbuilt statistical generator. Weekly drug treatments were as described above for xenograft assays.

Flow cytometry of mouse ccRCC tumours

ccRCC samples from Ksp1.3-CreER^{T2};*Vhl*$^{fl/fl}$;*Trp53*$^{fl/fl}$;*Rb1*$^{fl/fl}$ mice were minced with a scalpel in 5 ml HBSS at room temperature (RT), plates were washed with 40 ml HBSS and 5 ml of the 10x Triple Enzyme Mix (Hyaluronidase from bovine testes, Sigma-Aldrich, H3506, Collagenase from Clostridium histolyticum, Sigma-Aldrich, C5138 and Deoxyribonuclease I from bovine pancreas, Sigma-Aldrich, D5025-15KU) were added and the flask shaken at 80 rpm at RT for 1 hour. The solution was repeatedly pipetted up and down every 15 min. The cell suspension was centrifuged at 50 x g at RT for 10 min, the supernatant was collected and the bigger pellets on the bottom were discarded. The supernatant was centrifuged at 200 x g for 10 min and the pellet further washed in 10 ml wash buffer (1 g BSA + 372.24 g/mol EDTA in 1,000 ml HBSS) at 200 x g for 5 min. For this washing step the solution was moved to a 15 ml Falcon tube to avoid any loss of the pellet. The cell pellet was re-suspended in 1 ml ACK lysing buffer for around 1 min to deplete the red blood cells. After 1 min the ACK lysing buffer was diluted with 10 ml PBS and the tube centrifuged at 200 x g for 5 min. The pellet was re-suspended in MACS buffer (PBS 1x + 2 % FCS + 2 mM EDTA) and transferred into different wells of a 96-well plate. The cells in the 96-well plate were centrifuged at 1,600 rpm at 4 °C for 5 min and re-suspended in 50 µl LIVE/DEAD Fixable Aqua Dead Cell Stain (pre-diluted 1:500 in 1x PBS) to stain the dead cells positive. The cells were stained at 4 °C for 20 min and in the dark. Afterwards 120 µl MACS buffer was added to each well to dilute and wash away the LIVE/DEAD™ Fixable Aqua Dead Cell Stain and the 96-well plate was spun down at 1,600 rpm at 4 °C for 5 min. Subsequently, the pellets were re-suspended in 25 µl Fc Block (anti-mouse CD16/CD32, eBioscience/Thermo Fisher Scientific, 14-0161-85, 1:25 dilution) and incubated at 4 °C for 10 min and in the dark. After 10 min, 25 µl antibody mix was added additionally to the Fc Block solution and the cells incubated in the mix at 4 °C for 30 min and in the dark. The cells were stained with the following antibodies: anti-mouse CD45.2 APC-eFluor 780 (eBioscience, 47-0454-82, 1:100 dilution), anti-mouse CD3 Pacific Blue (BioLegend, 100214, 1:200 dilution), anti-mouse CD4 PerCP/C5.5 (BioLegend, 100434, 1:400 dilution), anti-mouse CD8a FITC (BD Biosciences, 561966, 1:200 dilution), anti-mouse CD279 (PD-1) PE-Cy7 (BioLegend, 109110, 1:400 dilution), anti-mouse CD45R/B220 Alexa Fluor 647 (BioLegend, 103226, 1:800 dilution), anti-mouse CD68 Brilliant Violet 605 (BioLegend, 137021, 1:400 dilution), anti-mouse F4/80 Alexa Fluor 488 (BioLegend, 123120, 1:200 dilution), anti-mouse/human CD11b Pacific Blue (BioLegend, 101224, 1:400 dilution), anti-mouse Ly-6G PE/Cy7 (BioLegend, 127618, 1:800 dilution), anti-mouse Ly-6C PerCP-Cy5.5 (BD Biosciences, 560525, 1:200 dilution), anti-mouse CD115 PE (BioLegend, 135505, 1:800 dilution). The cells were washed twice with MACS buffer and the intracellular staining was proceeded in specific wells. For the intracellular staining the pellets were re-suspended in 200 µl of FoxP3 Fixation/Permeabilization Buffer (Thermo Fisher Scientific, 00-5523-00, Fixation buffer:Permeabilisation buffer, 1:3 dilution) in which they were incubated at 4 °C for 30-60 min. Afterwards the cells were spun down and washed two times with 200 µl 1x Permeabilization Buffer (Thermo Fisher Scientific, 00-5523-00, 1:10 dilution in TE water). Further, the cell pellets were re-suspended in 100 µl 1x Permeabilization Buffer and 1 µl of anti-mouse/rat FoxP3 PE (eBioscience, 12-5773-82, 1:200 dilution) was added to each well. The cells were incubated in this antibody solution at RT for at least 30 min. Finally the cells were spun down again and washed two times with 200 µl 1x Permeabilization Buffer. After the two final washing steps the pellets were re-suspended in MACS buffer and the cells measured at the flow cytometer (BD LSRFortessa) with a 405 nm, 488 nm, 561 nm and 640 nm Lasers.

Statistics

The following statistical tests were employed in this study: Two-sided Student's unpaired t-test with Welch's correction, Running Enrichment Score with adjusted *P* values (padj) using the Benjamini-Hochberg correction, two-sided Mann-Whitney *U* tests without multiple comparisons, Fischer's exact test, non-parametric Kruskal-Wallis test with Dunn's correction for multiple comparisons, repeated measures analysis of variance (RM-ANOVA) followed by Tukey's post-hoc multiple pairwise comparisons, two-stage linear step-up procedure of Benjamini, Krieger and Yekutieli, Q = 1%, without assuming a consistent std. dev. between groups, Spearman correlation, Pearson correlation. $P < 0.05$ was considered statistically significant.

References

1. Harlander S, et al. Combined mutation in Vhl, Trp53 and Rb1 causes clear cell renal cell carcinoma in mice. *Nat Med* 2017;23(7):869–877.

2. Vichai V, Kirtikara K. Sulforhodamine B colorimetric assay for cytotoxicity screening. *Nat Protoc* 2006;1(3):1112–1116.

3. Tallarida RJ. Quantitative methods for assessing drug synergism. *Genes Cancer* 2011;2(11):1003–1008.

4. Frew IJ, et al. pVHL and PTEN tumour suppressor proteins cooperatively suppress kidney cyst formation. *EMBO J* 2008;27(12):1747–1757.

5. Gut G, Herrmann MD, Pelkmans L. Multiplexed protein maps link subcellular organization to cellular states. *Science* 2018;361(6401). doi:10.1126/science.aar7042

6. Bolger AM, Lohse M, Usadel B. Trimmomatic: a flexible trimmer for Illumina sequence data. *Bioinformatics* 2014;30(15):2114–2120.

7. Li H. Aligning sequence reads, clone sequences and assembly contigs with BWA-MEM2013;http://arxiv.org/abs/1303.3997. cited August 8, 2022

8. Wang K, Li M, Hakonarson H. ANNOVAR: functional annotation of genetic variants from high-throughput sequencing data. *Nucleic Acids Res* 2010;38(16):e164.

9. Cingolani P, et al. A program for annotating and predicting the effects of single nucleotide polymorphisms, SnpEff: SNPs in the genome of Drosophila melanogaster strain w1118; iso-2; iso-3. *Fly (Austin)* 2012;6(2):80–92.

10. Colaprico A, et al. TCGAbiolinks: an R/Bioconductor package for integrative analysis of TCGA data. *Nucleic Acids Res* 2016;44(8):e71.

11. Dobin A, et al. STAR: ultrafast universal RNA-seq aligner. *Bioinformatics* 2013;29(1):15–21.

12. Huber W, et al. Orchestrating high-throughput genomic analysis with Bioconductor. *Nat. Methods* 2015;12(2):115–121.

13. Grossman RL, et al. Toward a Shared Vision for Cancer Genomic Data. *N Engl J Med* 2016;375(12):1109–1112.

14. Luo W, et al. GAGE: generally applicable gene set enrichment for pathway analysis. *BMC Bioinformatics* 2009;10:161.

15. Ashburner M, et al. Gene ontology: tool for the unification of biology. The Gene Ontology Consortium. *Nat. Genet.* 2000;25(1):25–29.

16. Gene Ontology Consortium. Gene Ontology Consortium: going forward. *Nucleic Acids Res.* 2015;43(Database issue):D1049-1056.

17. Subramanian A, et al. Gene set enrichment analysis: A knowledge-based approach for interpreting genome-wide expression profiles. *Proceedings of the National Academy of Sciences* 2005;102(43):15545–15550.

18. Conway JR, Lex A, Gehlenborg N. UpSetR: an R package for the visualization of intersecting sets and their properties. *Bioinformatics* 2017;33(18):2938–2940.

19. Yu G, et al. DOSE: an R/Bioconductor package for disease ontology semantic and enrichment analysis. *Bioinformatics* 2015;31(4):608–609.

20. Barbie DA, et al. Systematic RNA interference reveals that oncogenic KRAS-driven cancers require TBK1. *Nature* 2009;462(7269):108–112.

21. Whitfield ML, et al. Common markers of proliferation. *Nat Rev Cancer* 2006;6(2):99–106.

22. Krug K, et al. A Curated Resource for Phosphosite-specific Signature Analysis. *Mol Cell Proteomics* 2019;18(3):576–593.

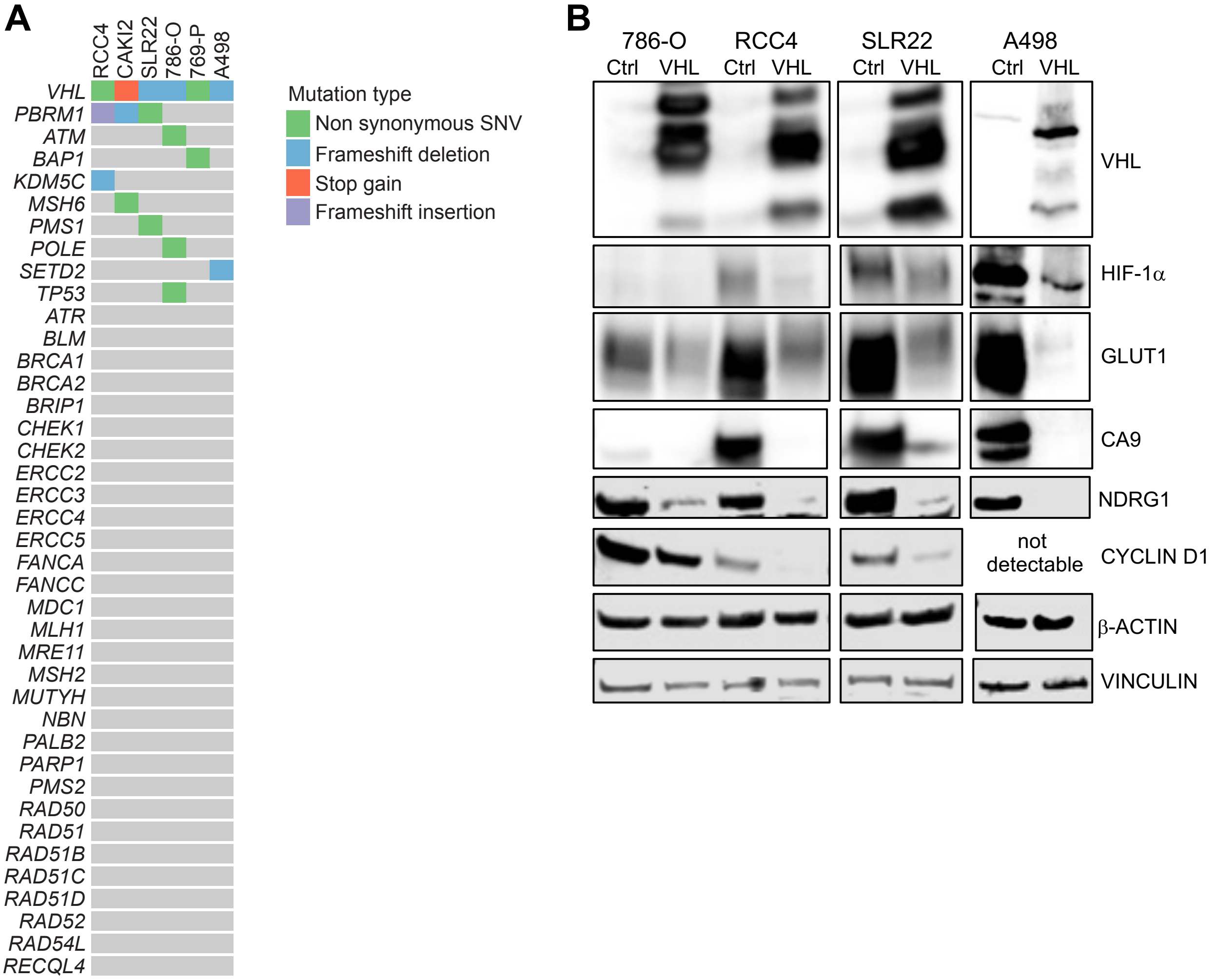

Supplementary Figure 1: Characterisation of ccRCC cell lines used in this study. **A.** Oncoprint showing mutations in epigenetic tumour suppressor genes and commonly mutated DNA damage related genes in the indicated ccRCC cell lines. **B.** Western blotting using antibodies against the indicated proteins in 786-O, RCC4, SLR22 and A498 cell lines infected with empty retroviral vector (Ctrl) or a vector expressing pVHL.

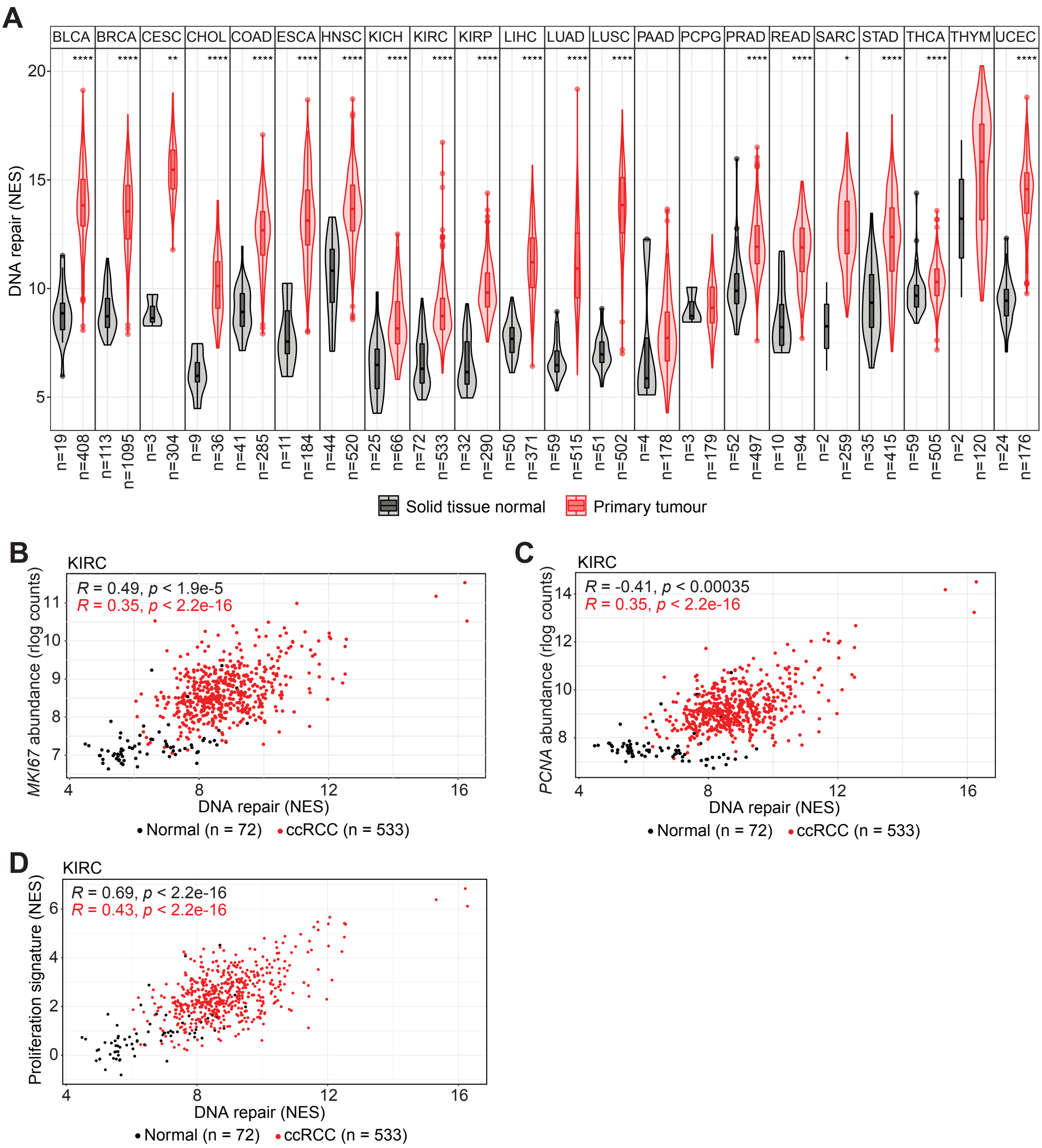

Supplementary Figure 2: Enrichment of Reactome DNA repair signature in other tumour types and correlation to proliferation in ccRCC. **A.** ssGSEA analysis of enrichment of the Reactome DNA repair signature in different TCGA tumour versus normal data sets. Violins show the kernel probability density of the NES deriving from the ssGSEA, boxes indicate the median and interquartile range (distance between 25th and 75th percentiles). Groups were compared using two-sided Mann-Whitney *U* tests without multiple comparisons (* *P* < 0.05, ** *P* < 0.01, *** *P* < 0.001, **** *P* < 0.0001). **B, C.** The correlation coefficient in the TCGA KIRC dataset between NES scores of the Reactome term DNA repair and the expression (rlog counts) of *MKI67* (**B**) or *PCNA* (**C**) was assessed using the Spearman correlation. **D.** The correlation coefficient in the TCGA KIRC dataset between NES scores of the Reactome term DNA repair and a proliferation signature was assessed using the Spearman correlation. The proliferation signature includes the genes *MKI67, PCNA, TOP2A, MCM2, MCM3, MCM4, MCM5, MCM6, PLK1, FOXM1, STK6* and *E2F1*.

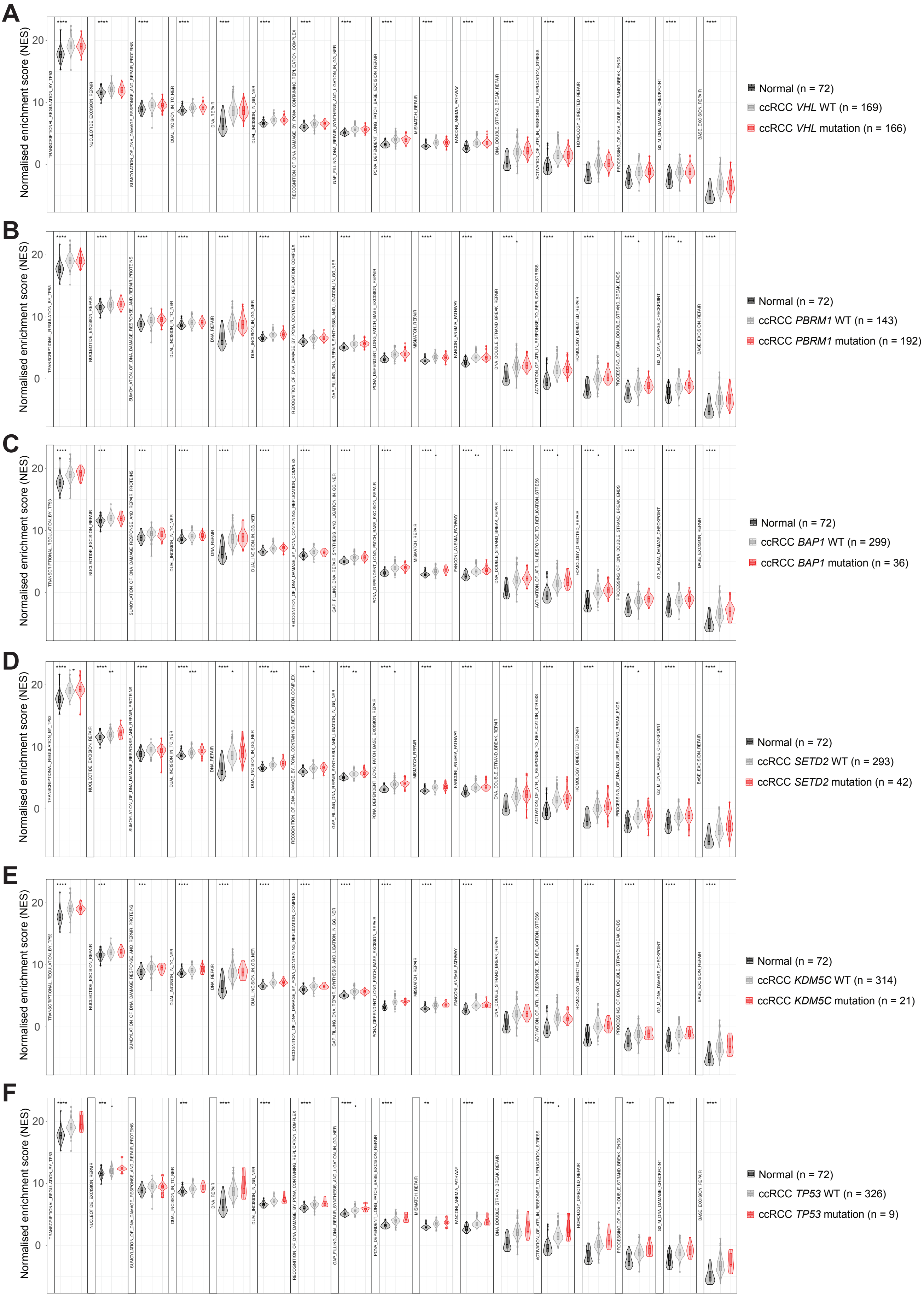
A
Normalised enrichment score (NES)
Normal (n = 72)
ccRCC VHL WT (n = 169)
ccRCC VHL mutation (n = 166)
B
Normalised enrichment score (NES)
Normal (n = 72)
ccRCC PBRM1 WT (n = 143)
ccRCC PBRM1 mutation (n = 192)
C
Normalised enrichment score (NES)
Normal (n = 72)
ccRCC BAP1 WT (n = 299)
ccRCC BAP1 mutation (n = 36)
D
Normalised enrichment score (NES)
Normal (n = 72)
ccRCC SETD2 WT (n = 293)
ccRCC SETD2 mutation (n = 42)
E
Normalised enrichment score (NES)
Normal (n = 72)
ccRCC KDM5C WT (n = 314)
ccRCC KDM5C mutation (n = 21)
F
Normalised enrichment score (NES)
Normal (n = 72)
ccRCC TP53 WT (n = 326)
ccRCC TP53 mutation (n = 9)

Supplementary Figure 3: Specific DNA damage gene expression signatures are enriched in tumours harbouring mutations in ccRCC tumour suppressor genes. **A-F.** Distribution of TCGA KIRC sample-specific normalised enrichment scores (NES) for specific Reactome pathways comparing normal tissue and ccRCC tumours with or without mutations in **A.** *VHL*, **B.** *PBRM1*, **C.** *BAP1*, **D.** *SETD2*, **E.** *KDM5C* and **F.** *TP53*. The violins show the kernel probability density of the NES deriving from the ssGSEA. The boxes indicate the median and interquartile range (distance between 25th and 75th percentiles). The tumour samples were split into samples with mutations in a specific epigenetic gene (red) and samples without mutations in the respective gene (grey). Two-sided Mann-Whitney *U* tests without multiple comparisons were performed to compare each group to the mutated samples (red). (* *P* value < 0.05, ** *P* value < 0.01, *** *P* value < 0.001, **** *P* value < 0.0001).

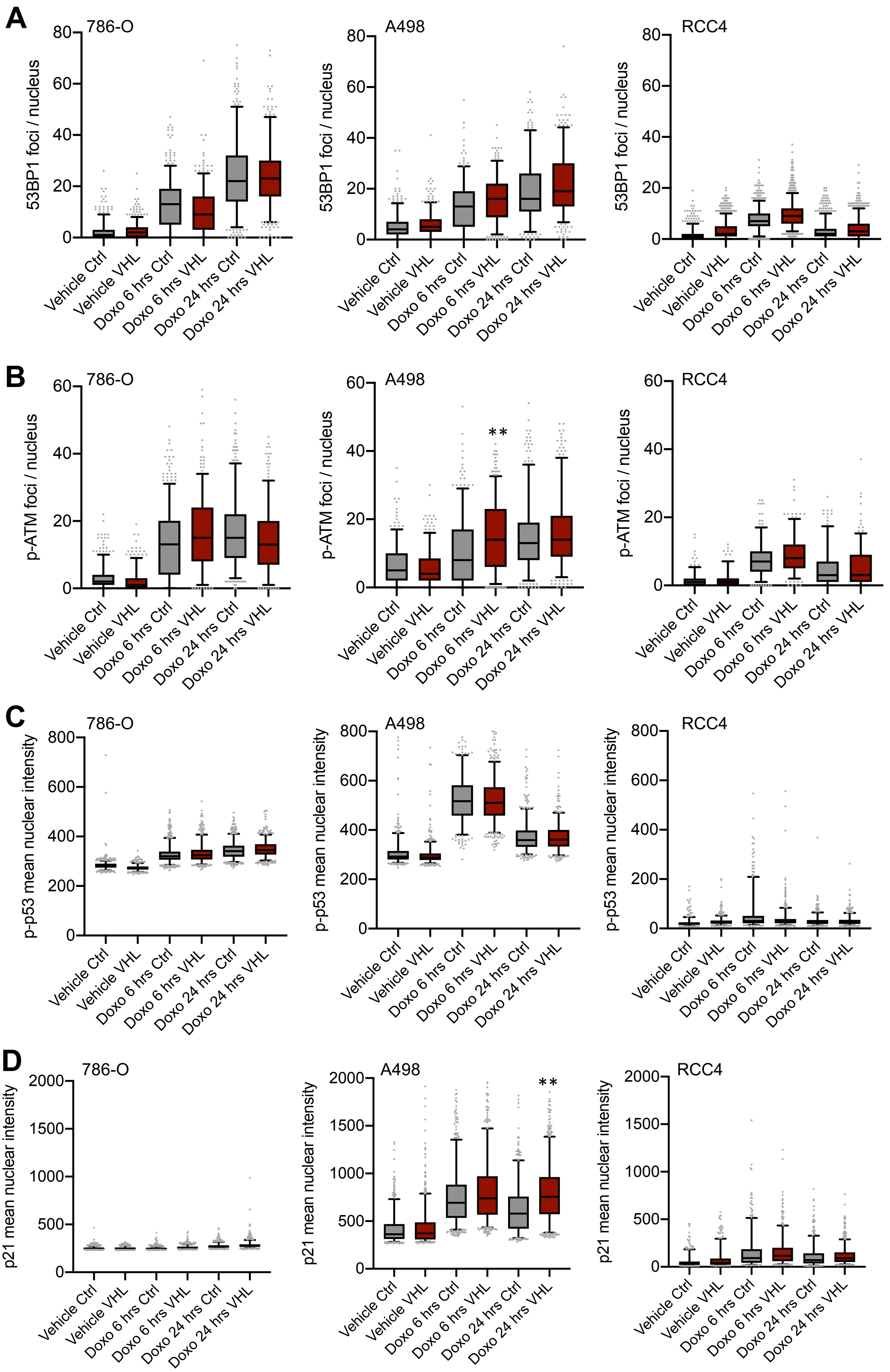

Supplementary Figure 4: pVHL restoration does not affect DNA damage induction or sensing in ccRCC cell lines. **A.** Quantification of nuclear 53BP1 foci. **B.** Quantification of nuclear p-ATM foci. **C.** Quantification of mean nuclear p-p53 intensity. **D.** Quantification of mean nuclear p21 intensity. Box plots show the quartiles, whiskers depict 5-95 percentiles and extreme values to the maximum and minimum are shown by grey dots. Statistical differences between doxorubicin treated versus control groups were assessed with the non-parametric Mann-Whitney test (** $P < 0.01$). Sample sizes range from 9 to 114 nuclei per condition.

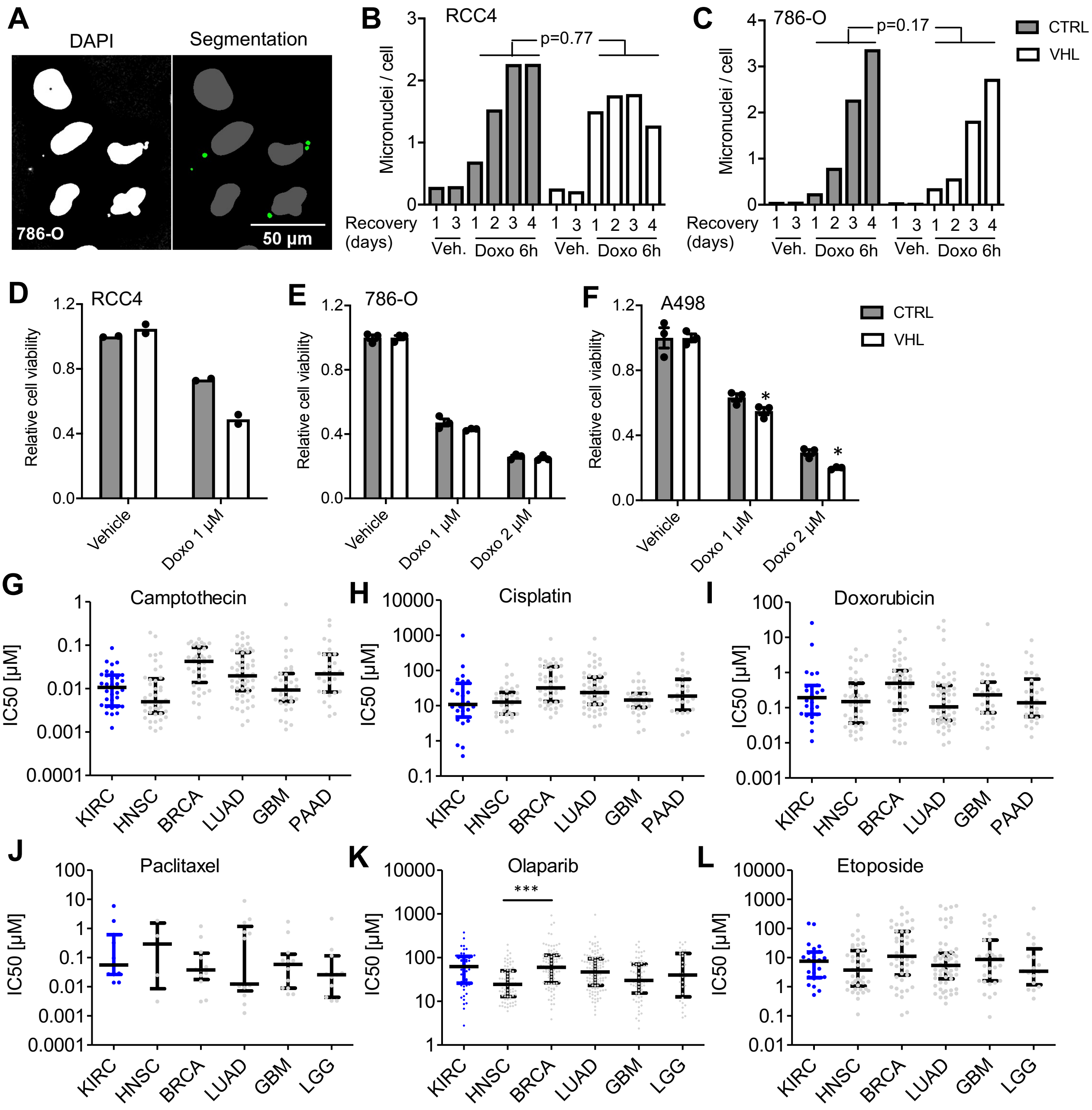

Supplementary Figure 5: ccRCC cells are sensitive to DNA damaging agents. **A.** Example of automated image segmentation to detect nuclei (grey) and micronuclei (green) based on DAPI staining. **B,C.** Quantification of micronuclei in RCC4 (**B**) and 786-O (**C**) cells expressing vector control or VHL reintroduction and treated with Doxorubicin for 6 hours followed by 1-4 days of recovery in the absence of drug. Bars represent the average number of micronuclei per nucleus determined from analyses of between 730 and 2649 nuclei per condition Student's t-test (two-sided, paired samples) showed no statistical difference in the kinetics of increase in micronuclei. **D-F.** Quantification of relative cell viability 6 days after treatment with Doxorubicin for 48 hours in the indicated ccRCC cell lines infected with empty retroviral vector (wt) or a vector expressing pVHL (VHL). Data represents mean or mean ± std. dev., independent experiments are shown as dots and two-sided *P* values were calculated by Student's t-test * $P < 0.05$. **G-L.** IC50 survival values from the "Genomics of Drug Sensitivity in Cancer Project" of renal carcinoma cell lines (KIRC) and cell lines from other human tumour types (HNSC: head and neck squamous carcinoma, BRCA: breast carcinoma, LUAD: lung adenocarcinoma, GBM: glioblastoma, PAAD: pancreatic adenocarcinoma) for the DNA damaging drugs camptothecin (**G**), cisplatin (**H**), doxorubicin (**I**), paclitaxel (**J**), olaparib (**K**) and etoposide (**L**). Each dot represents an independent cell line. Mean ± std. dev. are shown.

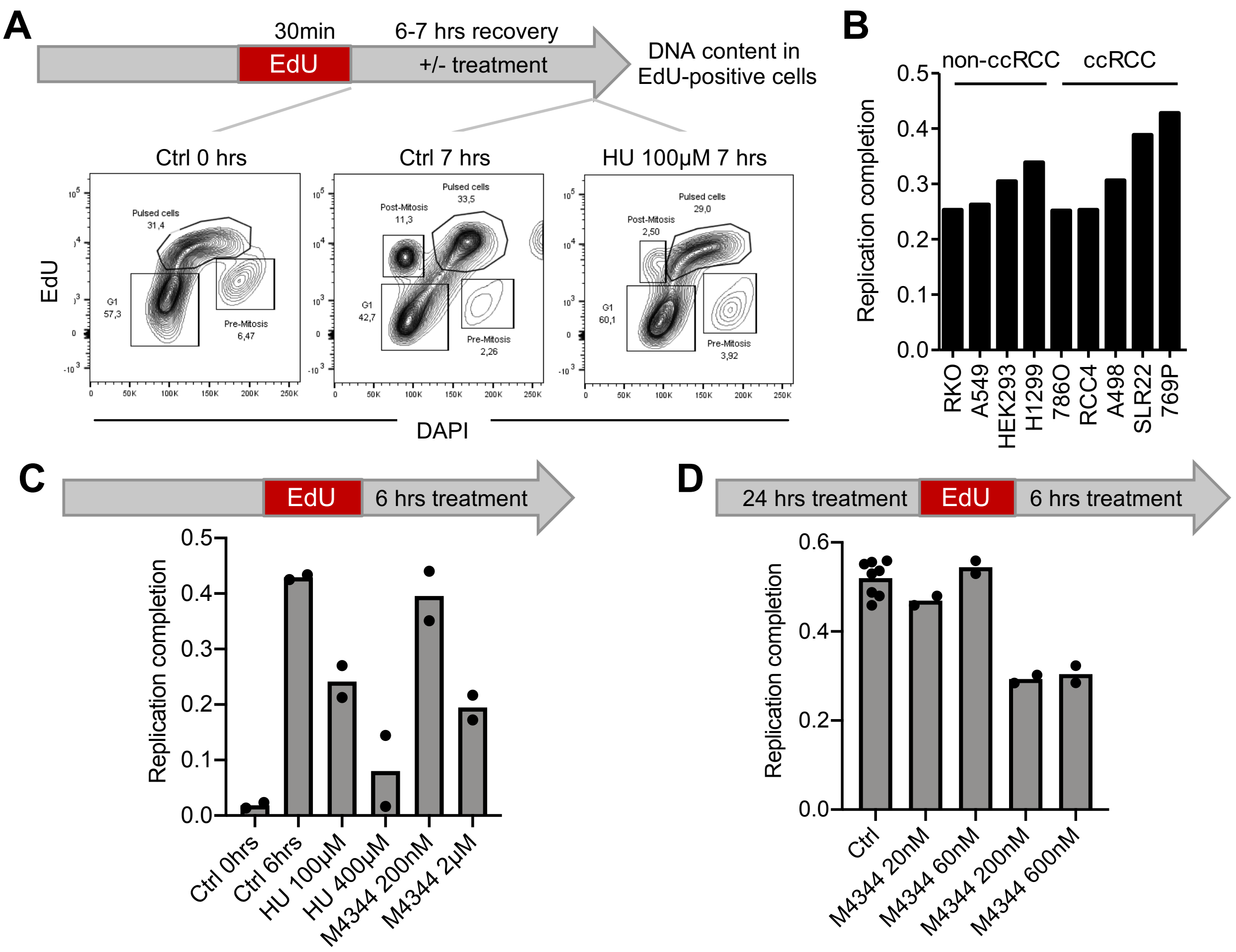

Supplementary Figure 6: M4344 delays S-phase progression. **A.** Overview of the FACS-based assay of S-phase progression and example of the effects of hydroxyurea (HU) on 786-O cells in this assay. **B.** Quantification of the relative S-phase progression rates in the indicated ccRCC and non-ccRCC cell lines. **C.** Quantification of the relative S-phase progression rates of 786-O cells treated with the indicated drugs for 6 hours after EdU pulse. **E.** Quantification of the relative S-phase progression rates of 786-O cells treated for 24 hours prior to, during and 6 hours after EdU treatment with the indicated drugs. Bars in C and D represent the mean values of independent experiments shown as dots.

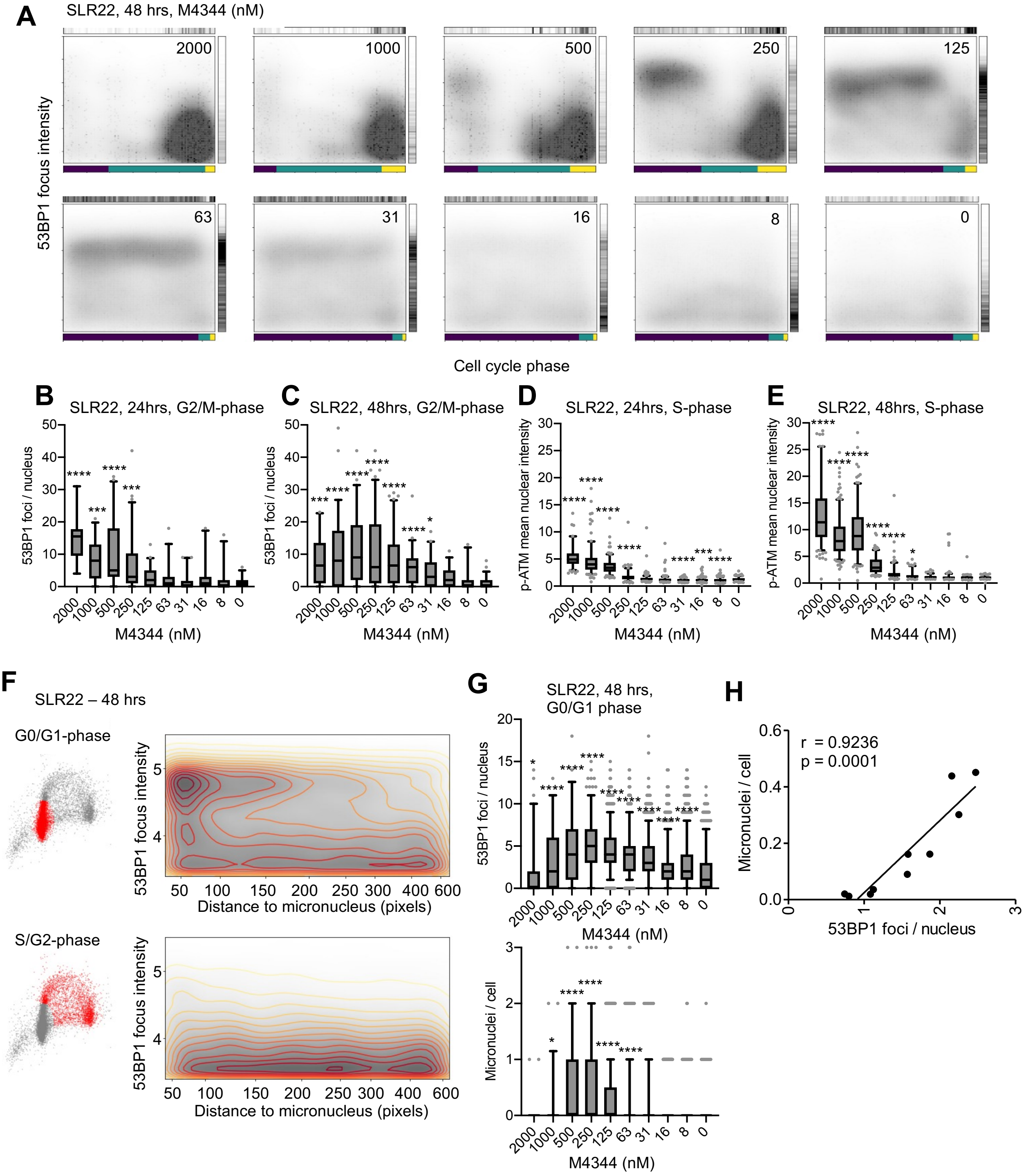

Supplementary Figure 7: Characterisation of mechanisms of DNA damage induced by M4344. **A.** 2D cell cycle resolution of 53BP1 focus intensities in SLR22 cells treated for 48 hrs with M4344. **B,C.** Quantification of 53BP1 focus intensities in G2/M-phase SLR22 cells treated for 24 hrs (**C**) or 48 hrs (**D**). **D,E.** Quantification of p-ATM mean nuclear intensities in S-phase SLR22 cells treated for 24 hrs (**C**) or 48 hrs (**D**). **F.** Density contour plots depicting distance from nucleus to micronucleus and 53BP1 focus intensity in G0/G1-phase or S/G2-phase SLR22 cells. **G.** Quantifications of 53BP1 foci per G0/G1 nucleus and number of micronuclei per GO/G1 nucleus 48 hours after treatment with the indicated concentrations of M4344. Box plots in B-E and G show the quartiles, whiskers depict 5-95 percentiles and extreme values to the maximum and minimum are shown by grey dots. Statistical differences between M4344 treated versus control groups were assessed with the non-parametric Kruskal-Wallis test with Dunn's correction for multiple comparisons (* *P* < 0.05, *** *P* < 0.001, **** *P* < 0.0001). Sample sizes range from 8 to 300 nuclei per condition (B-E) and 56-1512 nuclei per condition (G). **H.** Correlation between micronuclei and 53BP1 foci, Pearson correlation coefficient r and significance p are shown.

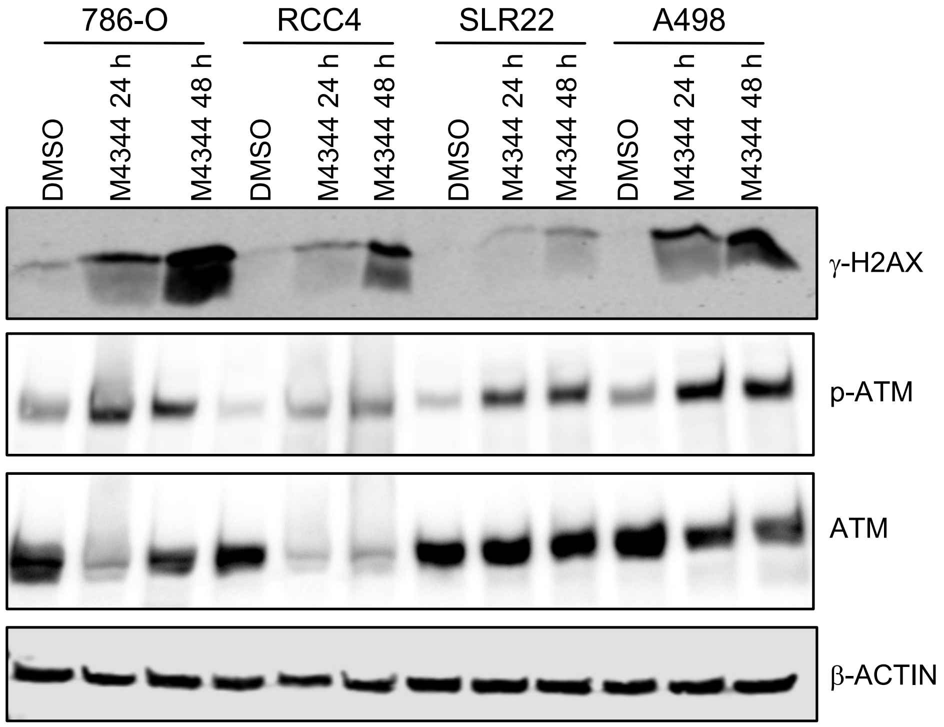

Supplementary Figure 8: M4344 induces DNA damage in ccRCC cell lines. Western blots of 786-O, RCC4, SLR22 and A498 cells treated for 24 or 48 hrs with 200 nM M4344 using antibodies against the indicated proteins.

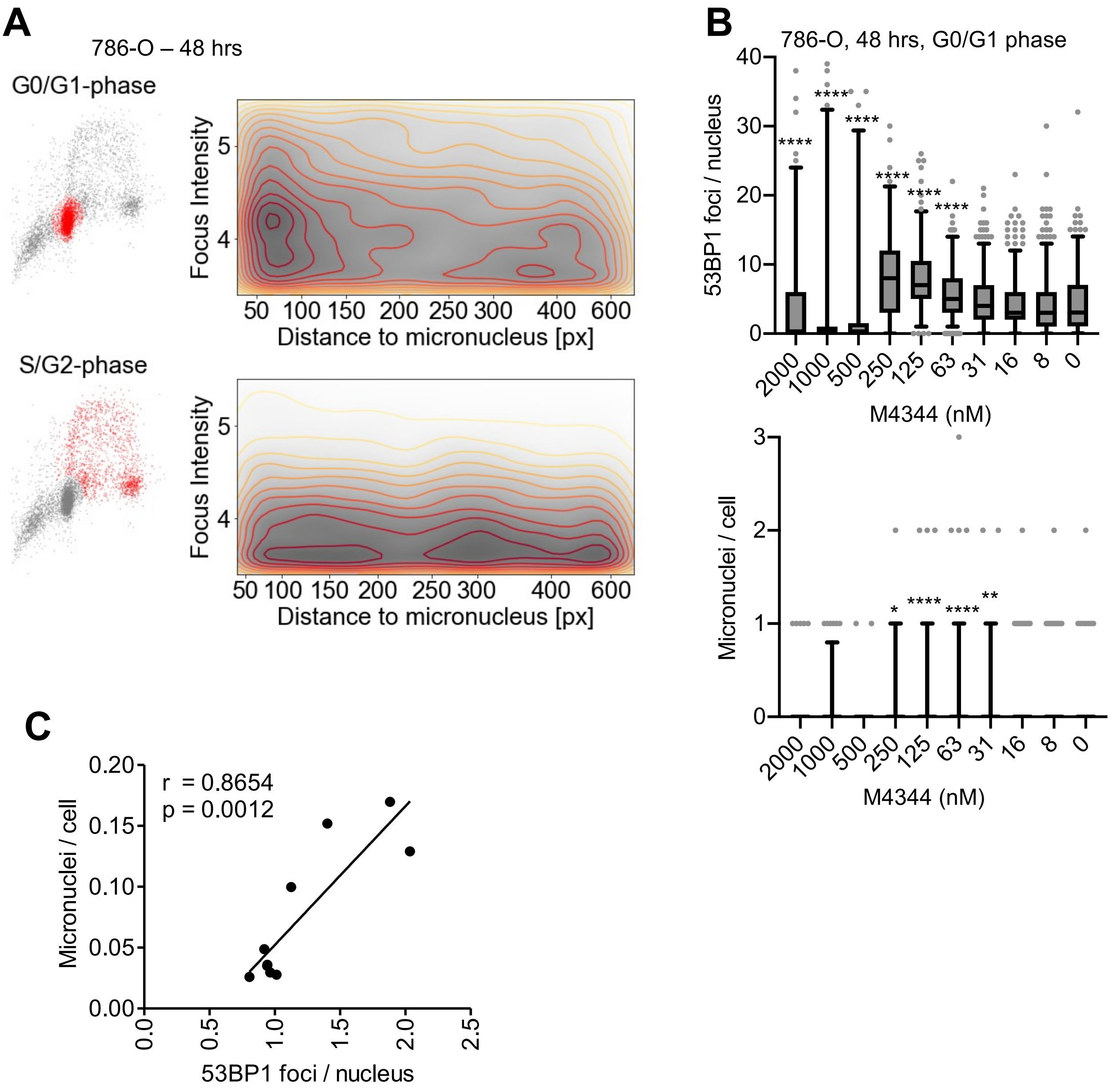

Supplementary Figure 9: M4344 induces formation of micronuclei. **A.** Density contour plots depicting distance from nucleus to micronucleus and 53BP1 focus intensity in G0/G1 phase or S/G2 phase 786-O cells. **B.** Quantifications of 53BP1 foci per G0/G1 nucleus and number of micronuclei per GO/G1 nucleus 48 hours after treatment with the indicated concentrations of M4344. Box plots show the quartiles, whiskers depict 5-95 percentiles and extreme values to the maximum and minimum are shown by grey dots. Statistical differences between M4344 treated versus control groups were assessed with the non-parametric Kruskal-Wallis test with Dunn's correction for multiple comparisons (* $P < 0.05$, ** $P < 0.01$, **** $P < 0.0001$). Sample sizes range from 77 to 371 nuclei per condition. **C.** Correlation between micronuclei and 53BP1 foci, Pearson correlation coefficient r and significance p are shown.

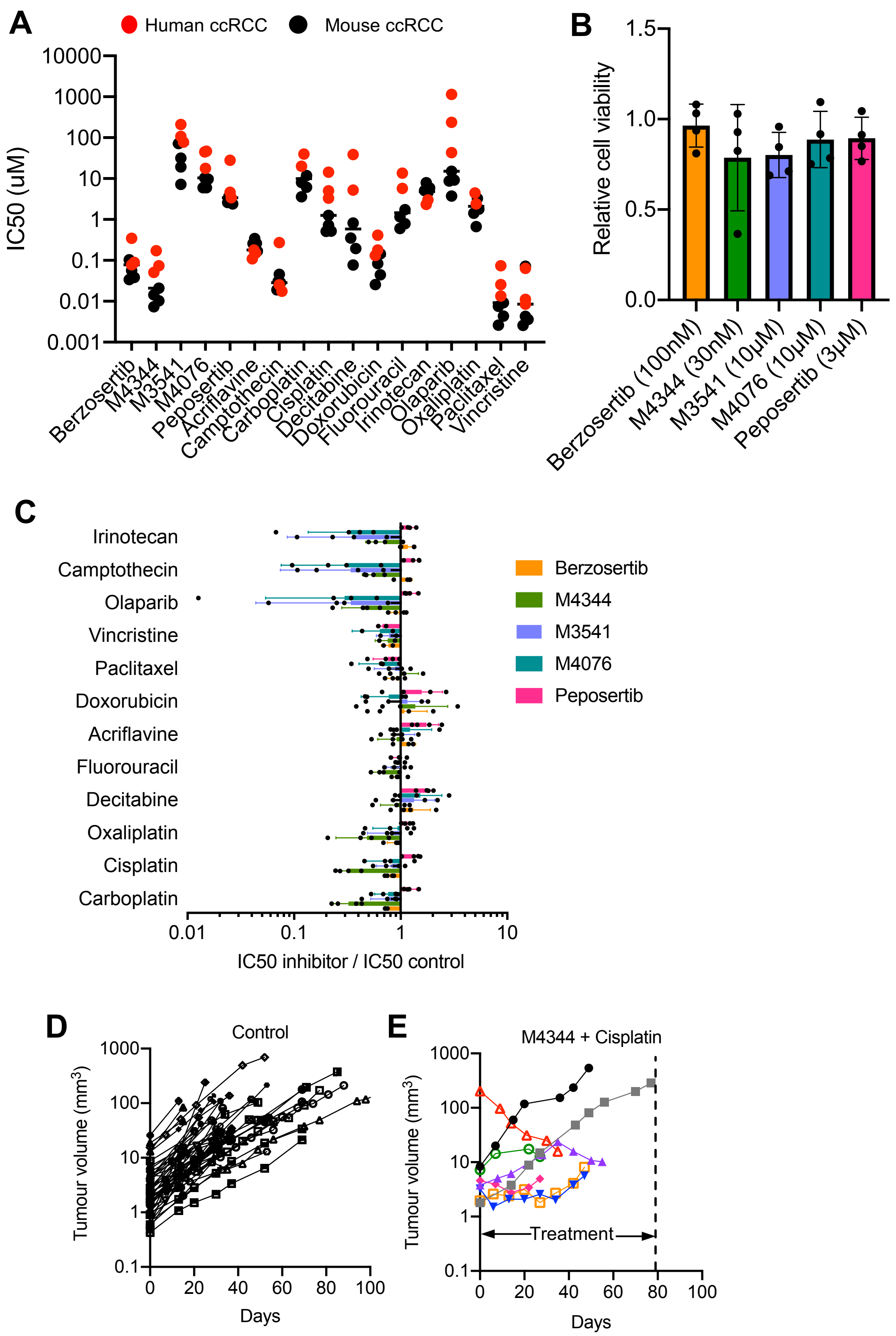

Supplementary Figure 10: *In vivo* therapeutic synergy between M4344 and cisplatin in *Vhl/Trp53/Rb1* mutant autochthonous ccRCC mouse model. **A.** IC50 cell viability values of four mouse *Vhl/Trp53/Rb1* mutant ccRCC cells (from Figure 1) and three human ccRCC cells (from Figure 3) in response to the indicated drugs. Each dot represents a different cell line. **B.** Effects of the indicated doses of ATMi, ATRi and DNA-PKi as single agents on cell viabilities of the four mouse ccRCC cell lines. **C.** Change in IC50 values (mean ± std. dev, four mouse ccRCC cell lines, each dot represents a different cell line) for each agent in the presence compared to absence of ATMi, ATRi and DNA-PKi drugs. **D,E.** *Vhl/Trp53/Rb1* mutant ccRCC tumour volumes over time in control mice (**D**) and M4344 + cisplatin treated mice (**E**). Each line represents an individual tumour.

Supplementary Figure 1

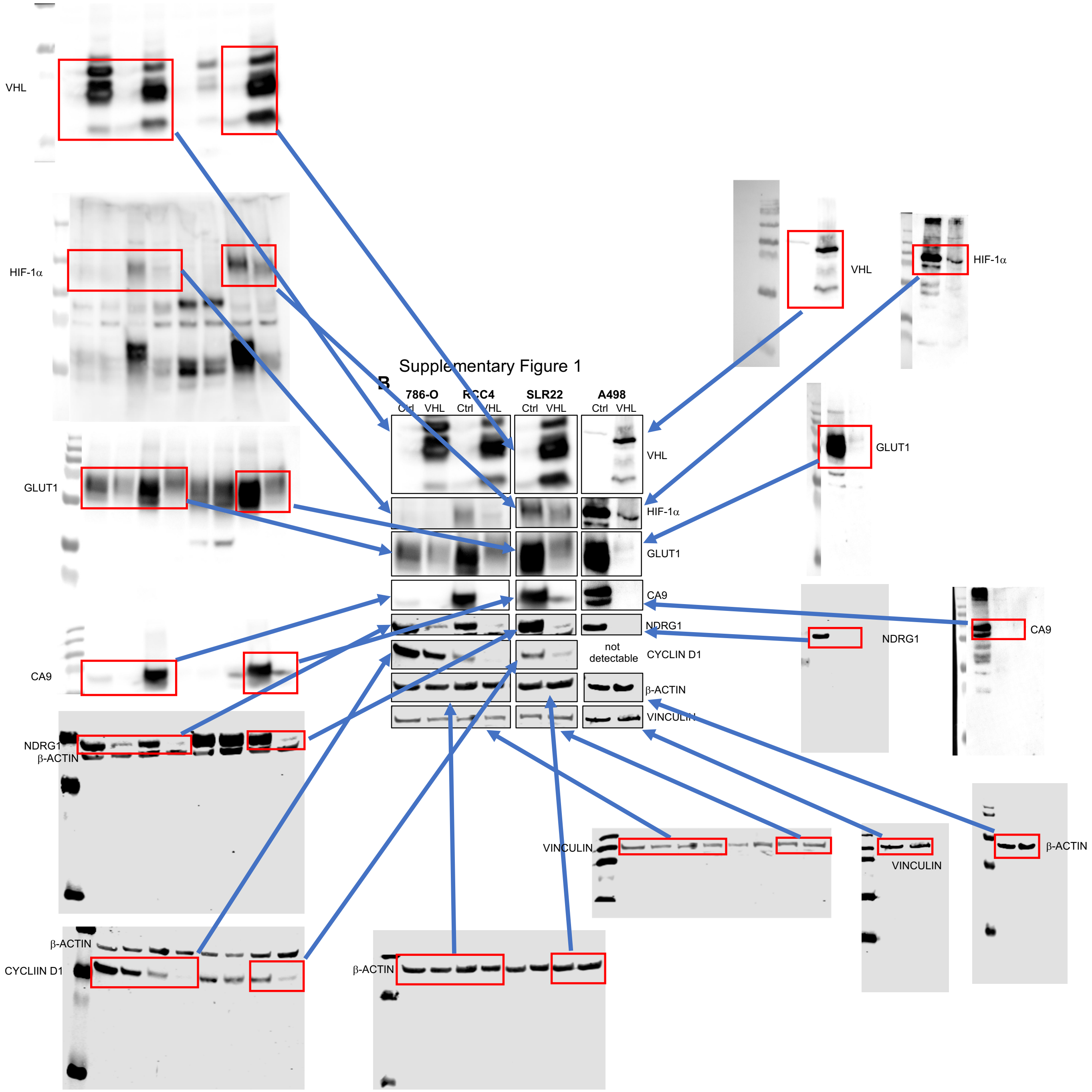

Supplementary Figure 8

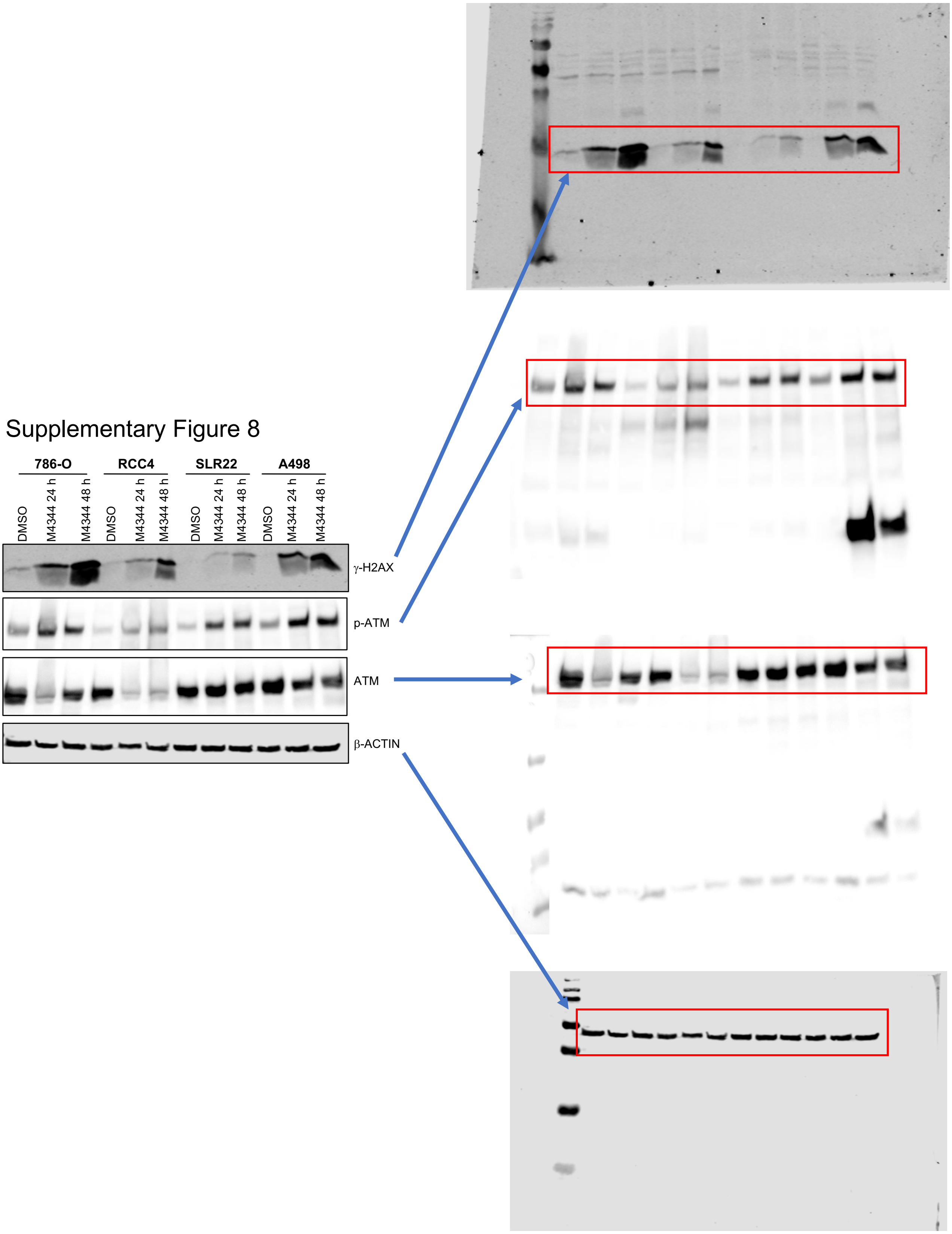

Supplement: Supplemental data [file jciinsight-7-156087-s057.pdf]
